# Effects of super-enhancers in cancer metastasis: mechanisms and therapeutic targets

**DOI:** 10.1186/s12943-024-02033-8

**Published:** 2024-06-07

**Authors:** Shenglan Liu, Wei Dai, Bei Jin, Feng Jiang, Hao Huang, Wen Hou, Jinxia Lan, Yanli Jin, Weijie Peng, Jingxuan Pan

**Affiliations:** 1 Key Laboratory of Prevention and Treatment of Cardiovascular and Cerebrovascular Diseases, Ministry of Education, Jiangxi Provincal Key Laboratory of Tissue Engineering, School of Pharmacy, Gannan Medical University, Ganzhou, 314000 China; 2 State Key Laboratory of Ophthalmology, Zhongshan Ophthalmic Center, Sun Yat-sen University, Guangzhou, 510060 China; 3 College of Public Health and Health Management, Gannan Medical University, Ganzhou, 341000 China; 4 College of Pharmacy, Jinan University Institute of Tumor Pharmacology, Jinan University, Guangzhou, 510632 China

**Keywords:** Super-enhancers, Metastasis, Molecular mechanisms, Therapeutic targets

## Abstract

Metastasis remains the principal cause of cancer-related lethality despite advancements in cancer treatment. Dysfunctional epigenetic alterations are crucial in the metastatic cascade. Among these, super-enhancers (SEs), emerging as new epigenetic regulators, consist of large clusters of regulatory elements that drive the high-level expression of genes essential for the oncogenic process, upon which cancer cells develop a profound dependency. These SE-driven oncogenes play an important role in regulating various facets of metastasis, including the promotion of tumor proliferation in primary and distal metastatic organs, facilitating cellular migration and invasion into the vasculature, triggering epithelial-mesenchymal transition, enhancing cancer stem cell-like properties, circumventing immune detection, and adapting to the heterogeneity of metastatic niches. This heavy reliance on SE-mediated transcription delineates a vulnerable target for therapeutic intervention in cancer cells. In this article, we review current insights into the characteristics, identification methodologies, formation, and activation mechanisms of SEs. We also elaborate the oncogenic roles and regulatory functions of SEs in the context of cancer metastasis. Ultimately, we discuss the potential of SEs as novel therapeutic targets and their implications in clinical oncology, offering insights into future directions for innovative cancer treatment strategies.

## Background

Today, most primary cancers can be cured by surgical resection and adjuvant treatment [1]. Despite surgical resection of primary cancer, metastasis still develops in 30–70% of patients, contributing to more than 90% of cancer-related deaths [2]. Systemic interventions, which include screening, targeted therapies, chemotherapy, and immunotherapy, have demonstrated efficacy in both the prevention and management of metastasis, leading to significant improvements in patient outcomes. Notably, the 3-year relative survival rate for lung cancer has increased by 11.0%, and that for metastatic melanoma has increased by 18.7% [3]. Despite these advances in oncological treatment, mortality rates continue to rise for certain cancer types, including those of the liver, pancreas, uterus, and various sarcomas. Further, the majority of patients diagnosed with recurrent or *de novo* metastatic cancer still face a poor prognosis, with most succumbing to the disease within 5 years [4]. One of the substantial hurdles in advancing cancer treatment is the incomplete understanding of the oncogenes driving metastasis. This knowledge gap has limited the development of targeted therapeutic strategies. Consequently, preventing or eradicating metastases remains a formidable challenge.

Metastasis is a process in which tumor cells spread from the primary sites to other parts of the body via lymphatic and/or blood circulation [5]. This phenomenon entails a sophisticated sequence of cellular and biological events that can be summarized as follows: (1) In the primary tumor, cancer cells undergo epithelial-mesenchymal transition (EMT), leading to diminished cell–cell adhesion, thereby fostering local migration and invasion; (2) these cells then intravasate into the lymphatic and/or bloodstream by degrading the extracellular matrix (ECM), transitioning into circulating tumor cells (CTCs); (3) once in circulation, CTCs develop stemness characteristics, enabling them to resist anoikis, evade immune detection, and overcome shear stress, thus ensuring their survival in the bloodstream; (4) subsequently, these cells extravasate into distant organs and tissues; and (5) upon reaching a new site, the cells adapt to the local tumor microenvironment, initiate colonization, promote angiogenesis, and finally, outgrow at the metastatic sites, including but not limited to the liver, lung, brain, and bone [5, 6]. The successful completion of each step in this cascade is imperative for the formation of metastases, whereas failure in either of these processes can inhibit the progression of metastasis. A better understanding of the biological function and vulnerabilities in the metastatic cascade provides opportunities to unveil novel targets that could be exploited for medical therapy.

Alterations in transcriptional regulation resulting from genetic or epigenetic modifications are fundamental for endowing primary cells with the capability to metastasize [7, 8]. Investigations conducted by Roe et al. and Teng et al., which involved comparative analyses between metastatic and respective primary samples, elucidated that the mechanism propelling metastasis predominantly involves epigenetic reprogramming over genetic alterations [7, 9]. Super-enhancers (SEs), representing a critical axis of epigenetic regulation, consist of extensive clusters of regulatory DNA elements that significantly orchestrate gene transcription [10]. In comparison to typical enhancers (TEs), SEs exhibit an approximately tenfold increase in the density of transcription factors (TFs), transcriptional regulators, active histone modifications, co-activators, chromatin regulators, and RNA Polymerase II (Pol II) [11, 12]. Consequently, SEs not only actively promote the transcription of exon-encoded genes but also drive the expression of noncoding RNAs (ncRNAs) [13].

SEs are not only crucial for determining the identity of somatic cells but also play a significant role in the advancement of cancer [14]. Recent research has identified SEs across various cancer subtypes, revealing their role in driving the prolific expression of critical oncogenic genes or ncRNAs [15, 16]. These SE-driven oncogenes or ncRNAs are essential in modulating cellular phenotypes, including EMT, migration, invasion, stemness, and angiogenesis—traits that facilitate the dissemination of tumor cells to distant organs, leading to the formation of metastases [17–19]. Further investigations have shown that these SE-associated genes or ncRNAs rely heavily on sustained active transcription, allowing for targeted effects even before the occurrence of global suppression of transcriptional activity [20, 21]. This unique dependency of the SE-driven transcriptional program has provided new therapeutic targets for the treatment of cancer metastasis. In this review, we describe the formation, biogenesis, activation, and identification methods of SEs, along with delineating their pivotal roles and the underlying molecular mechanisms in cancer metastasis. Furthermore, we explore emerging pharmacological inhibitors that target SEs, discussing their potential in advancing cancer therapy.

## Discovery history and properties of SEs

**Fig. 1:**
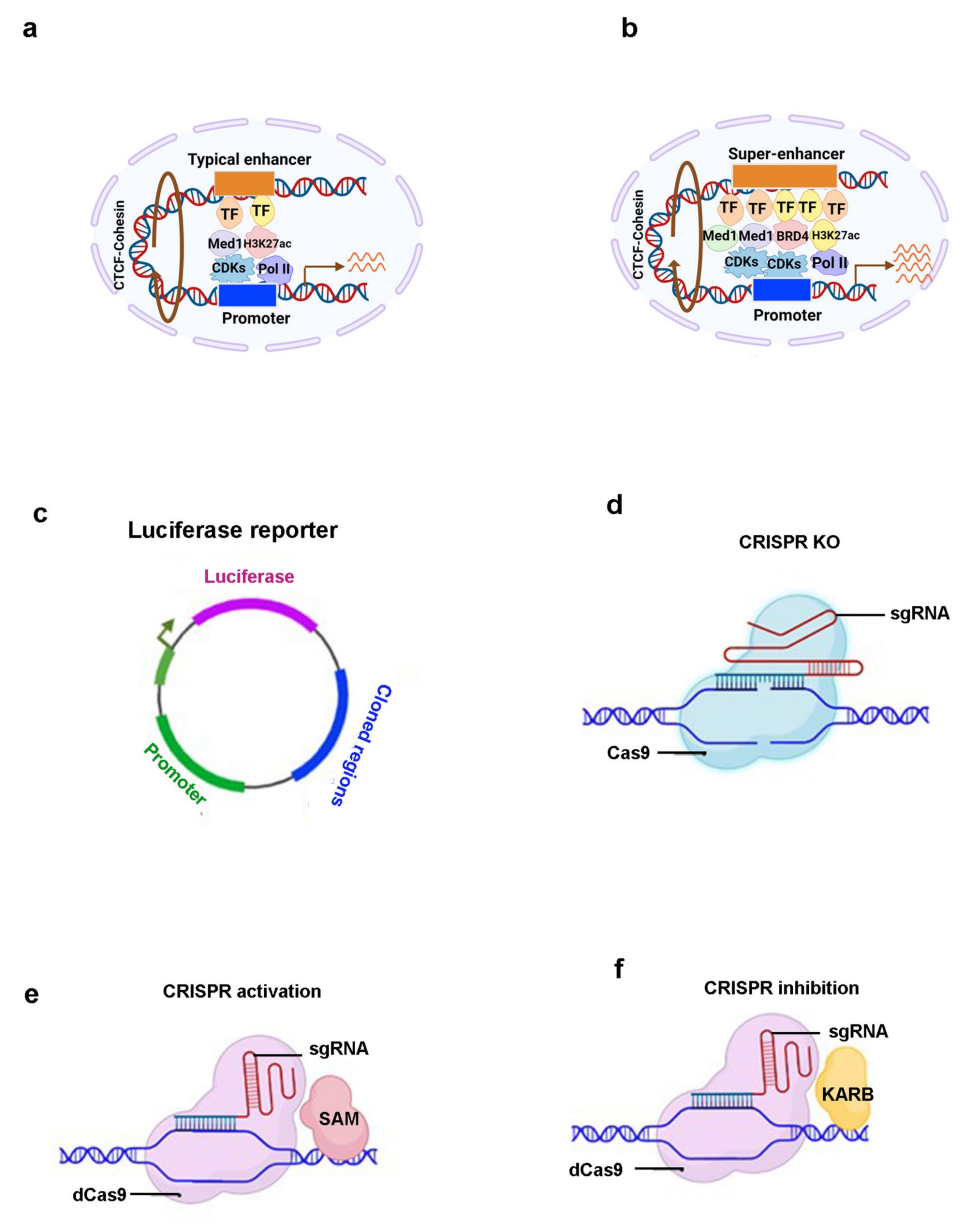
Structure and activity verification methods of super-enhancers (SEs). **a** Typical enhancers are DNA elements bound by transcription factor (TFs) that recruit a moderate amount of H3K27ac, BRD4, CDKs, Med1 and RNA polymerase II (Pol II), which contributes to the expression of target genes at normal levels. **b** SEs possess the same components as typical enhancers, yet in significantly higher density, consequently facilitating vigorous transcription of target genes. The stability of enhancer-promoter interaction is stabilized through the binding of CTCF and cohesin. **c** Selected enhancer regions within SE are cloned into a reporter vector. Subsequently, the enhancer activity is vitrificated through a dual luciferase reporter assay after constructing the plasmid. **d** CRISPR/Cas9 constructs containing specific small guide RNAs (sgRNAs) targeting the enhancer region are constructed to assess the function of SE by individually depleting each element. **e** The CRISPR activation system utilizes the catalytically deactivated Cas9 (dCas9) and sgRNAs to guide transcriptional activators (such as VP64 or the histone acetyltransferase p300) to specific genomic sites within SEs, facilitating effective transcriptional activation and enabling the assessment of SE activity. SAM, synergistic activation mediator. **f** The CRISPR inhibition complex contains the dCas9 and sgRNAs to guide transcriptional repressors (e.g. the KRAB repressor protein or histone demethylase LSD1, DNMT3A) to specific genomic loci, facilitating robust transcriptional suppression and allowing for the evaluation of SE activity

Researchers have been investigating the regulation of transcription by enhancers since the 1980s [22]. Enhancers are short regulatory DNA sequences ranging from 100 to 300 bp. Regardless of the distance, location, or orientation, enhancers can increase promoter activity by recruiting specific TFs to the transcription start sites (TSS), thereby driving the expression of target genes (Fig. 1a). The human genome contains hundreds of thousands of enhancers [23, 24]. Subsequent investigations into enhancer-mediated transcriptional regulation mechanisms have revealed the existence of sophisticated, multi-component transcriptional regulators. These regulators are characterized by a wide array of mechanistic features, including aggregated *cis*-regulatory elements, locus control regions, and transcription initiation zones, further demonstrating the complexity and diversity of transcriptional control mechanisms [25].

In 2013, Young’s group reported a large cluster of adjacent enhancers spanning several kilobases within 12.5 kilobases (kb), which determine the identity of embryonic stem cells (ESCs) [14]. These unusual enhancers are called SEs, which are *cis*-regulatory regions of genes that are densely occupied by Mediator complex and master regulators (Fig. 1b). SEs, which are structurally akin to TEs, are positioned in close proximity to the promoters of target genes through chromatin looping facilitated by the CCCTC-binding factor (CTCF) and the cohesin complex, thereby robustly promoting transcription [26]. Distinct from TEs, SEs are characterized by several key differentiators: (1) their larger DNA size; (2) denser accumulation of master TFs (e.g. Oct4, Sox2, Nanog) and associated co-factors such as Mediator Subunit 1 (Med1) and p300, alongside active histone modifications (e.g. histone H3 lysine 4 monomethylation [H3K4me1], histone H3 lysine 27 acetylation [H3K27ac]), chromatin modulators (e.g. Bromodomain-containing protein 4 [BRD4], cyclin-dependent kinase 7 [CDK7]), and RNA Pol II; (3) an enhanced capacity to drive transcription; and (4) increased vulnerability to perturbation of SEs [27, 28]. These distinctions underscore the important role of SEs in the regulation of gene expression, particularly in contexts that are critical for maintaining and establishing cellular identity.

The advancement of technologies for the comprehensive screening and identification of SEs has significantly accelerated in recent years, where a suite of specialized methods has been developed, predominantly using ChIP-seq as the foundational technique (Table 1). Such examples are chromatin interaction analysis with paired-end tag (ChIA-PET) sequencing [29], chromatin immunoprecipitation sequencing (PLAC-seq) [30], Hi-C on Accessible Regulatory DNA (HiCAR) [31], ChIP-exo [32], Hi-ChIP [33], ChIA-Drop [34], and Cleavage Under Targets and Tagmentation (CUT&Tag) [35]. These approaches facilitate the exploration of interactions within SE regions mediated by histone modifications, TFs, or cofactors. Moreover, the integration of ChIP-seq with techniques that discern the three-dimensional (3D) chromatin architecture, such as chromosome conformation capture (3 C) and its derivatives: 4 C (circular chromosome conformation capture), 5 C (chromosome conformation capture carbon copy), also enhances our ability to analyze interactions between SE regions and gene promoters. In addition, sensitive methodologies such as the assay for transposase-accessible chromatin using sequencing (ATAC-seq), DNase-seq, micrococcal nuclease sequencing (MNase-seq), formaldehyde-assisted isolation of regulatory elements sequencing (FAIRE-seq), and self-transcribing active regulatory region sequencing (STARR-seq) are employed to verify the increased accessibility associated with SEs. These approaches facilitate more accurate, systematic, and comprehensive investigations into the contributions of SEs to physiological and pathological processes. Further, the advent of online databases dedicated to SEs offers invaluable resources for SEs prediction (Table 2). These databases provide annotations on the potential roles of SEs in gene transcriptional regulation and the modulation of regulatory networks across various biological processes. This convergence of advanced technologies and resources significantly enhances our understanding of the intricate mechanisms underlying SE-mediated genes and their implications in health and disease.

**Table 1:** Approaches for identifying super-enhancers (SEs)

Methods	Description	Advantages	Disadvantages	Required number of cells
ChIP-seq	A tool for identifying the DNA regions bound by a specific protein of interest	High resolution, low noise, and large genome coverage	High cost for library construction and critically on the quality of the antibody	10^7^~10^8^
ChIP-exo	A tool for mapping an interest protein-binding DNA locations with lambda exonuclease digestion (exo)	High resolution, low background, and precise at near-base pair resolution	More expensive, complex, and technically challenging	10^7^~10^8^
ChIA-PET	A tool for studying chromatin interactions mediating by a specific protein of interest	Identify both proximal and distal 3D chromatin interactions at the whole-genome scale	The sequencing platform is expensive and takes a long to run	10^7^~10^8^
Hi-ChIP	A tool for examining the regulatory interactions between distal DNA elements modulating by a protein of interest	Few cells required, low noise, and high efficiency	Only capture DNA interactions mediated by a specific target protein	10^5^~10^6^
ChIA-Drop	A tool for investigating multiplex chromatin interactions regulating by a protein of interest in single-molecule resolution	Few cells required, simple, stable and high efficiency	Raw sequencing reads are huge and difficult to map and analyze	10^3^~10^4^
PLAC-seq	A tool for identifying certain protein-mediated chromatin interactions	High resolution and accuracy, fast, sensitive, and high efficiency	Only detect chromatin sequences that are associated with specific proteins	10^6^~10^8^
HiCA	A tool for analyzing open chromatin anchored interactions	Low-input cells, high-resolution, sensitive, and cost-effective	Less sensitive to capture weak open chromatin peaks	10^4^~10^6^
CUT&Tag	A tool for the detecting DNA regions bound by a specific protein of interest with the enzyme-tethering strategy	High resolution, low background and small amount of starting material	Not very suitable for analyzing genome regions that are silent or contain heterochromatin	10^0^~10^4^
ATAC-seq	A tool for assessing regions of open chromatin, providing insights into gene regulation and accessibility	Fast, sensitive and a small amount of sample	High background noise and sensitive to experimental conditions	10^0^~10^5^
DNase-seq	A tool for identifying regions of open chromatin across the genome by sequencing DNA that is accessible to DNase I enzyme digestion	High resolution and precise	Large amounts of starting material and the results can vary depending on the DNase I digestion efficiency	10^6^~10^8^
MNase-seq	A tool for mapping of nucleosome positioning and chromatin structure across the genome	High-resolution and accurate	Biases due to differential accessibility of regions to micrococcal nuclease	10^6^~10^7^
FAIRE-seq	A tool for identifying regions of open chromatin	Simpler and less expensive	High background noise and lower resolution	10^6^~10^7^
STARR-seq	A tool for quantitatively assessing the enhancer activity across the genome	Directly measure the enhancer activity	Large amounts of input DNA, high complexity and technical demands	10^7^~10^8^
Luciferase reporter assay	A tool for measuring the activity of the regulatory sequence by linking it to a reporter gene	Sensitive, quantitative and high-resolution	Require careful control of experimental variables to avoid artifacts	10^3^~10^5^
CRISPR/Cas9	A tool for precise alterations to the DNA sequence at specific sites, enabling targeted gene modification	High specificity and efficiency	Sometimes introduce off-target effects or mutations at non-target sites	10^3^~10^5^
CRISPRa	A tool for utilizing a modified CRISPR/Cas9 system paired with transcriptional activators to selectively enhance gene expression	High specificity and efficiency	Off-target effects	10^3^~10^5^
CRISPRi	A tool for utilizing a modified CRISPR/Cas9 system paired with transcriptional repressors to selectively inhibit gene expression	High specificity and efficiency	Off-target effects	10^3^~10^5^

ATAC-seq: accessible chromatin using sequencing; ChIA-PET: chromatin interaction analysis with paired-end tag; ChIP-seq: chromatin immunoprecipitation sequencing; CRISPRa: CRISPR activation; CRISPRi: CRISPR inhibition; CUT&Tag: Cleavage Under Targets and Tagmentation; FAIRE-seq: formaldehyde-assisted isolation of regulatory elements sequencing; HiCAR: Hi-C on Accessible Regulatory DNA; PLAC-seq: chromatin immunoprecipitation sequencing; STARR-seq: self-transcribing active regulatory region sequencing

**Table 2:** Online available databases of SEs

Database	Description	Website
SEdb	Provides source for SEs annotation and potential functions 9 in human and mouse	https://bio.liclab.net/sedb/
SEA	Provides information for SEs, Typical enhancers (TEs), SNPs, transcription factor binding sites, chromatin interactions and Cas9 target sites in 11 species, including zebrafish, chicken, human, mouse and so on	http://sea.edbc.org/
Cistrome Cancer	Provides comprehensive resource for predicted enhancer profiles, SE-associated genes, and transcription factor targets in cancers	http://cistrome.org/CistromeCancer/
FANTOM5	Provides information for transcriptional regulatory network, predicted enhancer regions and SE profiles in human	https://slidebase.binf.ku.dk/human_enhancers/

## Identification methods of SEs

Next-generation sequencing (NGS) technologies alongside high-throughput sequencing are employed to identify SEs. The identification process relies on chromatin immunoprecipitation sequencing (ChIP-seq) to gauge the enrichment of master TFs and cofactors, including Med1, BRD4, H3K27ac, H3K4me1, and p300. Following this, the identified enhancers are combined and subjected to a hierarchical ranking process. Subsequently, the Rank Ordering of Super Enhancers (ROSE) algorithm, pioneered by Young and colleagues, is predominantly employed. This algorithm is often used to separate SEs from TEs on the basis of their respective ChIP-seq signal intensities [12]. Further, for a more refined identification of SEs, the Hypergeometric Optimization of Motif Enrichment (HOMER) bioinformatics tool is employed, offering a sophisticated analysis that corroborates the identification of SEs within genomic data [36].

After the identification of SEs utilizing the methods discussed above, it becomes imperative to empirically test, validate, and quantify their activity through functional assays. These methods include the following: (1) reporter assays [37, 38], (2) CRISPR/Cas9-mediated genetic perturbation [39], (3) CRISPR activation (CRISPRa), (4) CRISPR inhibition (CRISPRi) approaches [40, 41] (Table 1). To elucidate the potential synergistic, additive, or complex effects amongst SE constituents, Hnisz et al. employed a luciferase reporter assay. This approach involved integrating the SE element into a reporter vector to assess their individual and combined effects (Fig. 1c). Their findings revealed distinct activity levels for each component within the *Pou5f1* SE region, suggesting that these elements do not necessarily act in a synergistic or additive manner in ESCs [39]. In addition, the CRISPR/Cas9 system has been used to excise specific segments within an SE to determine their individual contributions to gene regulation, both in vitro and in vivo (Fig. 1d). For example, research in leukemia demonstrated that the SEs adjacent to the *MYC* gene comprised multiple enhancer modules, each exhibiting unique activity levels. By employing dual sgRNAs in conjunction with Cas9 mRNA to systematically delete these elements, it was revealed that MYC expression is governed by the cumulative and coordinated activity of these enhancer modules [42]. Additionally, CRISPRa and CRISPRi strategies have been adopted to modulate the transcriptional activity of specific SE components. These methods utilize a catalytically deactivated Cas9 (dCas9) to direct either transcriptional activators (e.g. VP64 or the histone acetyltransferase p300) or repressors (e.g. KRAB repressor protein or the histone demethylase LSD1, DNMT3A) to each targeted SE loci, thereby assessing the transcriptional outcome and functional role of each SE constituent (Fig. 1e-f) [18]. A notable study by Dai et al. in colorectal cancer (CRC) used the CRISPRa system to target SE elements of CCDC137, significantly augmenting both the transcriptional activity and function of CCDC137. Conversely, employing CRISPRi to target the SE elements of CCDC137 markedly reduced its expression and functional impact [8]. This suite of functional assays facilitates a comprehensive understanding of the role and dynamics of SE elements in gene regulation, offering insights into their contribution to cellular identity and disease mechanisms.

## Classification of SEs

SEs profoundly enhance the transcription of genes in both physiological and pathological states by establishing a complex interaction network with promoters. This network comprises TFs, Mediator complex, cofactors,

, SEs, and the target genes themselves [43]. Notably, SE regions are occupied with diverse TFs, including master TFs which possess the unique capability to modulate their own expression levels. This self-regulatory mechanism lays the foundation for a core transcriptional regulatory circuitry (CRC) [44]. In the context of ESCs, key transcriptional programs are driven by master TFs such as OCT4, SOX2, and NANOG. These factors localize to their respective SE regions, forming intricate autoregulatory loops. Such circuits are important in fine-tuning the expression of pivotal genes, thus maintaining the ESC identity [12]. Similarly, in cancer cells, proteins like ELF3, MYCN, and TGIF1 engage in comparable self-sustaining loops within SE domains, propelling the transcription of essential oncogenic TFs and their associated genes [45–47]. The CRC model facilitates a deeper comprehension of the role of SEs in cell type-specific transcriptional regulation. By perpetuating self-reinforcing feedback mechanisms, SEs ensure the persistent expression of key regulatory genes, playing a critical role in sustaining distinct cellular identities and functions across various biological contexts and conditions.

Beside exon-encoded genes, SEs also promote the expression levels of ncRNAs to regulate biological functions, including enhancer RNA (eRNAs) [15], long noncoding RNAs (lncRNAs) [48], microRNAs (miRNAs) [49], and circular RNAs (circRNAs) [50]. eRNAs, for example, are transcriptional outputs of SE domains, typically ranging from 0.5 to 5 kb in length and are considered a subset of lncRNAs [51]. Research has elucidated that eRNAs contribute to gene expression regulation through facilitating SE-promoter looping interaction. They play significant roles in orchestrating various biological processes associated with tumorigenesis, such as initiation, proliferation, adhesion, apoptosis, migration, and immune modulation [15, 52]. Further, a plethora of other ncRNAs regulated by SEs, including miRNAs [53], circRNAs [54], and lncRNAs [48], have been implicated in promoting tumor progression and metastasis. They achieve these effects primarily through the indirect modulation of oncogenic signaling pathways, unveiling the multifaceted roles of SEs in the regulation of gene expression and their profound impact on cancer biology.

## Formation of SEs

Extensive genome-wide analyses have elucidated that SEs are acquired through various mechanisms, including (1) genetic mutation, (2) 3D chromatin changes, (3) viral infections, and (4) abnormal transactivation and oncogenic signaling. These findings underscore the multifaceted origins of SEs, highlighting their complexity and the broad spectrum of factors that contribute to their formation and evolution within the genomic landscape.

### Genetic mutation

**Fig. 2:**
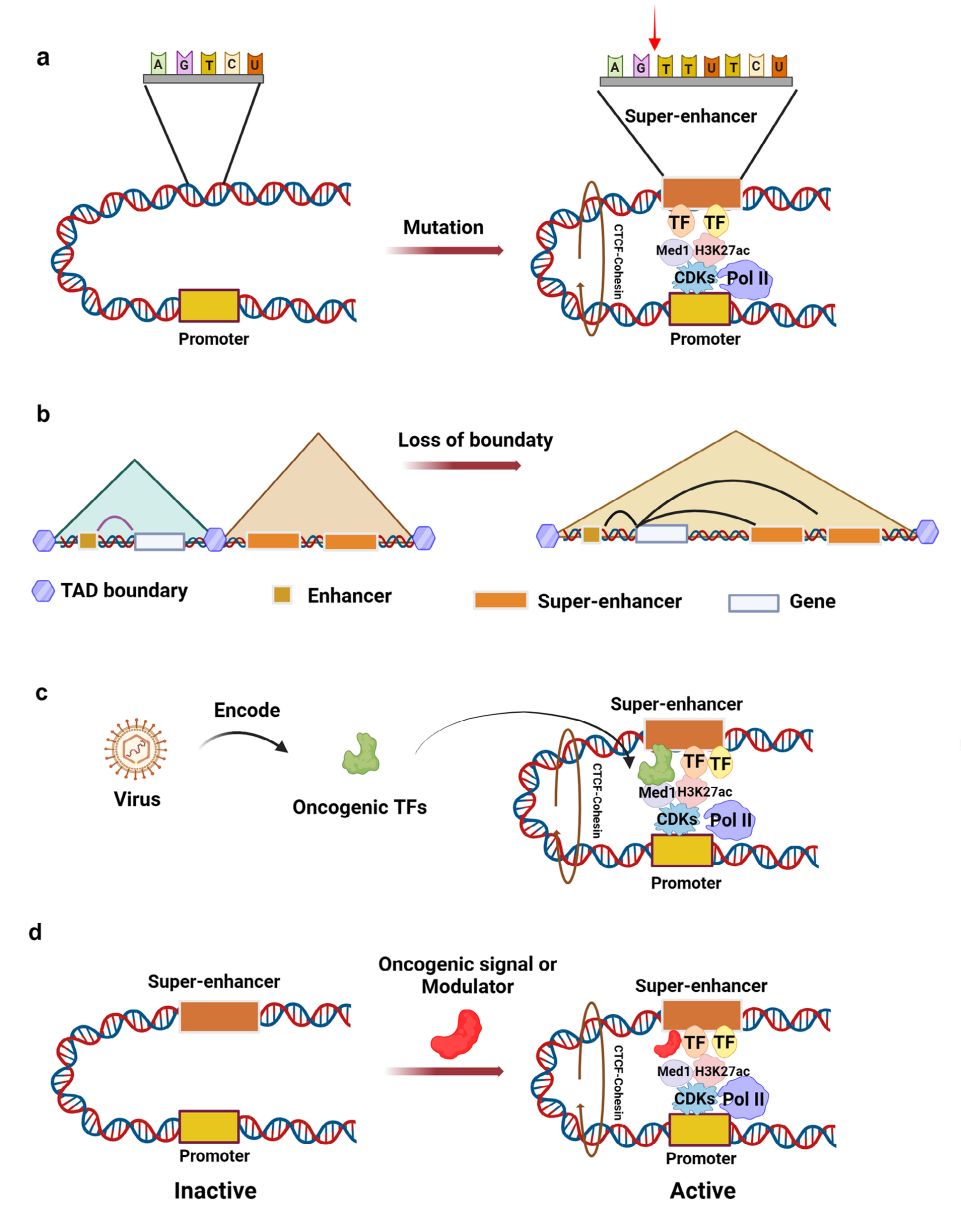
Mechanisms responsible for the formation of SEs. **a***De novo* generation of TF binding sites through genomic rearrangements, sequence insertions/deletions, or translocations in the genome. **b** Structural variations or epigenetic dysregulation can modify the three-dimensional chromatin structure, causing abnormal interactions between SEs and promoters, consequently enhancing the expression of nearby oncogenes. **c** Specific proteins or TFs encoded by viruses (such as EBV, HTLV-I) promote the formation of SEs in infected cells. **d** Persistently active signaling pathways trigger the activation of effective TFs to bind to susceptible genomic regions, initiating the generation of SEs

Genetic alterations in regulatory regions, such as genomic copy number changes, rearrangements, sequence insertions/deletions (indel), translocations, or single-nucleotide polymorphisms (SNPs), can modify SE landscapes to cause aberrant target gene expression (Fig. 2a). These genetic variations lead to SE activation or repression, resulting in abnormal expression of nearby target genes. Copy number gains creating SEs proximal to oncogenes is a common mechanism that promotes cancer pathogenesis. Integrative analysis of somatic copy number and epigenomic profiles has revealed that focal amplification of noncoding regions leads to oncogenic SE formation and activation of driver transcription programs across multiple cancer types [55]. In addition, SNPs disrupting TF binding sites can dysregulate oncogenic SE transcriptional output. An example is the LMO1 SNP rs2168101 G > T in neuroblastoma, which induces addiction to LMO1 expression [56]. Further, somatic insertions can create *de novo* TF binding sites that nucleate new SE formation and inappropriate gene induction. For example, small noncoding insertions introducing MYB motifs upstream of *TAL1* gene in patients with T-cell acute lymphoblastic leukemia (T-ALL) were found to generate a TAL1-activating SE through MYB and cofactor recruitment [57]. Thus, genetic alterations reprogramming SE landscapes represent a common contributor to cancer transcriptional dysregulation.

### Three-dimensional chromatin changes

Topologically associating domains (TADs) are megabase-sized structural units formed by chromatin looping that spatially constrain enhancer-promoter contacts [58]. Mammalian genomes are partitioned into TADs within which frequencies of interactions are enriched compared to those between TADs [59]. However, genetic or epigenetic alterations disrupting TAD boundaries can aberrantly fuse domains and rewire regulatory chromatin interactions to promote cancer progression (Fig. 2b). For instance, eliminating CTCF insulator sites at TAD borders in lung cancer enabled the spreading of active chromatin to a merged TAD, forming an IRS4 SE that drove oncogenic IRS4 overexpression [60]. In addition, IDH mutations in gliomas were found to cause CTCF binding site hypermethylation, leading to reduced CTCF binding and disruption of TAD borders disruption. This permitted aberrant activation of *PDGFRA* gene, a prominent glioma oncogene, by a potent enhancer that was previously isolated from the gene’s promoter [61]. Thus, genetic or epigenetic perturbations dismantling TAD organization can elicit pathogenic gene misexpression via illicit enhancer-promoter contacts. Therefore, preservation of TAD integrity is critical for constraining transcriptional noise during cell state transitions relevant to cancer emergence.

### Viral infections

Infection by various viruses, such as Epstein–Barr virus (EBV), human T-cell leukemia virus type 1 (HTLV-I), and human hepatitis B virus, constitutes an additional pathway for SE formation, which promotes high-level transcription of critical oncogenic genes (Fig. 2c). In the context of adult T-cell leukemia/lymphoma (ATLL), significant collaboration between viral and cellular TFs has been documented in SE development. Mechanistically, the HTLV-I-encoded essential TF, HBZ, has the capability to interact with the SE domain of *BATF3*, thus initiating the transcription of BATF3 and its downstream targets, which are instrumental in leukemic transformation [62, 63]. Further, multiple investigations have shown that EBV synthesizes oncoproteins (e.g. EBNA2, 3 A, 3 C) that interact with NF-κB subunits, leading to the establishment of around 200 SEs. These SEs are pivotal in driving the transcription of genes that essential for cell survival and anti-apoptotic functions, including MYC and BCL2, thereby facilitating the proliferation of lymphoblastoid cells [64–66].

### Abnormal trans-acting signaling pathway

Only a small fraction of these critical oncogenic SEs is established as a result of *cis*-acting mechanisms. A majority of SEs are established without alteration of the chromatin structure, which indicates that *trans*-acting mechanisms may contribute to SE formation (Fig. 2d) [67]. Research has elucidated that TFs, responding to oncogenic cues such as WNT, leukemia inhibitory factor (LIF), and transforming growth factor (TGF)-β, bind to specific DNA recognition motifs, thereby inducing the development of SEs [39, 68, 69]. Hnisz et al. observed that following Wnt stimulation, the terminal TF TCF4 localized to the SE at the *c-MYC* locus, a well-known target of Wnt signaling in CRC. This SE activity within the *c-MYC* region diminished upon disruption of the Wnt pathway [39]. These findings underscore the hypothesis that the dysregulation of oncogenic signaling pathways may facilitate the acquisition of SEs by oncogenes within tumor cells. Similarly, in breast cancer, estrogen receptor alpha (ERα) binds to multiple SE sites in response to estrogen signaling, a phenomenon that is absent in normal breast epithelium [70].

In addition to oncogenic signaling, chromatin regulators acting as *trans*-activating factors also play roles in SE formation. SWI/SNF complexes are ATP-dependent nucleosome remodeling enzymes, which belong to an important category of chromatin remodelers [71]. Shi et al. discovered that SWI/SNF complexes binding at distal enhancers cluster at the *MYC* gene site to create hyperactive SEs, leading to enhanced chromatin looping interactions, increased TF occupancy, and upregulated ncRNA transcription in acute leukemia [72]. In addition, BRD4, an essential epigenetic regulator that recognizes acetylated lysine on histones, modulates the chromatin landscape and recruits other TFs to gene promoters. In the context of foam cells, the activity of BRD4 is necessary for SEs of the proinflammatory cytokines IL-1β and IL-8. The disruption of BRD4 function using JQ1 or siRNAs impedes the association between the IL-1β promoter and its SE elements, and also disrupts the interaction between the IL-8 promoter and its corresponding SE elements [73].

## Activation of SEs

The liquid-liquid phase separation (LLPS) model has recently been proposed to elucidate the mechanisms underlying SE assembly and activation [67]. Since SEs represent hubs of high-density interactions between transcriptional modulators and nucleic acids, they can rapidly form these membrane-less, phase-separated organelles [74]. These structures quickly exchange components within the intracellular context to activate robust transcription in response to environmental cues [75]. Supporting this model, Sabari et al. demonstrated that transcriptional coactivators such as BRD4 and Med1 occupy SE regions, forming phase-separated droplets mediated by the intrinsically disordered regions (IDRs) of BRD4 and Med1 [28]. Interestingly, the Med1-IDR, rather than the BRD4-IDR, facilitates the compartmentalization and condensation of the transcriptional apparatus at specific SE-associated genes. Similarly, SE-bound TFs such as OCT4 and GCN4 can also form phase-separated droplets with Mediator to promote gene activation [76]. Further, Jeong et al. reported that IDRs contained within NUP98-HOXA9, a TF chimera, are required for establishing LLPS puncta to drive the oncogenic gene-expression program by forming broad SE-like binding patterns [77]. By condensing the transcriptional machinery, SE-based micelles may explain the abundant transcription levels, the rapid nucleation and specific susceptibility to perturbations. Mutations in critical IDRs or the depletion of associated proteins or DNA regions significantly decrease transcription and dissociate other SE components.

## Roles and mechanisms of SEs in metastasis

**Fig. 3:**
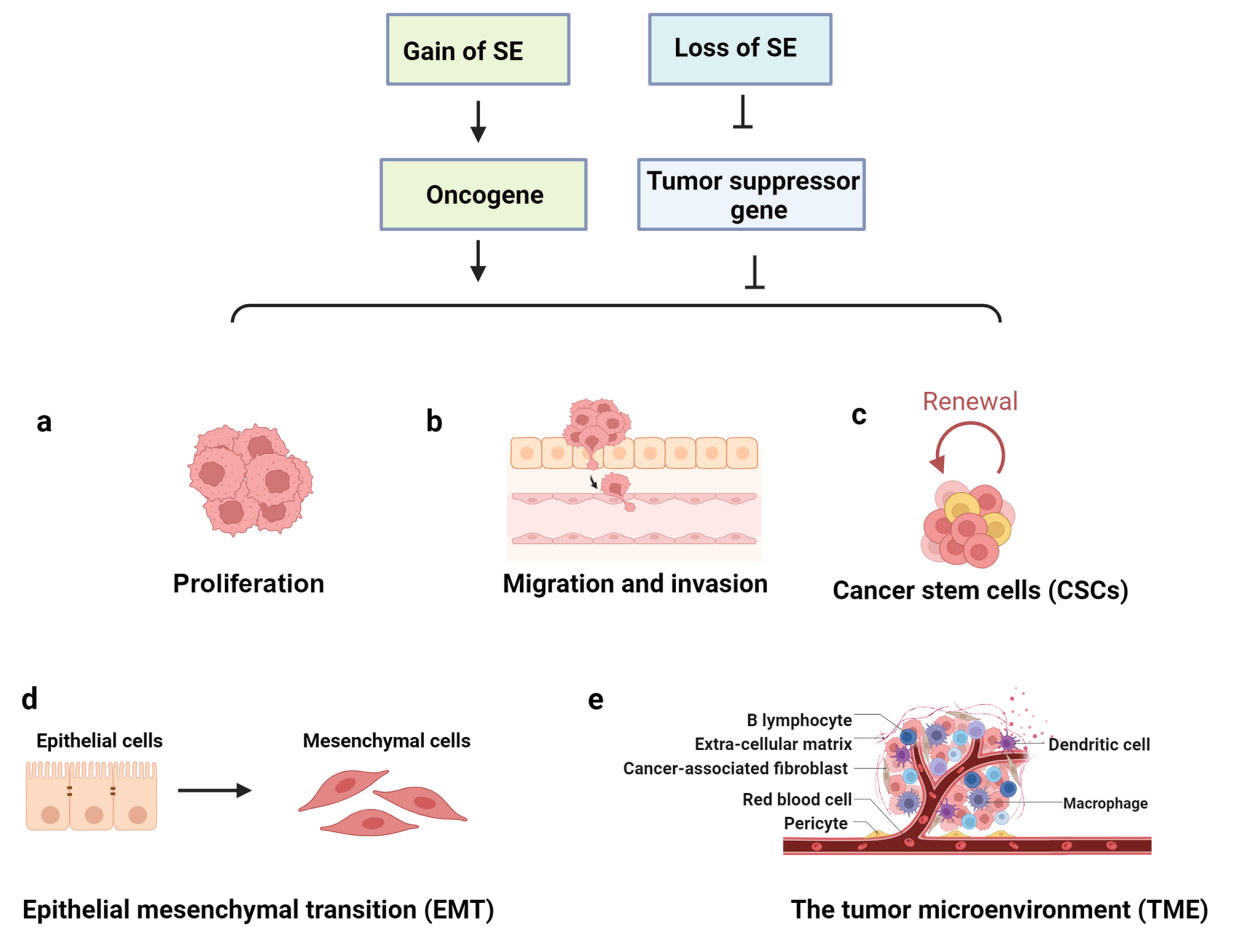
Gain or loss of SEs enhances malignant tumor metastasis through either upregulating oncogene expression or downregulating tumor suppressor gene expression. SEs can foster metastasis-related cellular characteristics by modulating key molecular factors associated with proliferation, migration, invasion, cancer stem cells (CSCs) formation, epithelial-mesenchymal transition (EMT), and the tumor microenvironment (TME)

Tumor metastasis is a dynamic process in which malignant cells spread to target tissues and organs from the original tumor location, where their persistent proliferation causes secondary tumors along the way [13]. However, no particular driver gene unique to metastases has been found [78]. NGS data indicate that epigenetic alternation is probably a critical mechanism for facilitating metastasis because no specific mutation has been observed between primary and metastatic pancreatic tumors [79]. Further, research has demonstrated that SE, as a vital epigenetic regulation mechanism, plays important roles in cancer metastasis [9, 80, 81]. In the following sections, we explore the potential relationships between SE-mediated regulatory mechanisms and metastasis, including (1) proliferation, (2) EMT, (3) migration and invasion, (4) cancer stem cells (CSCs), and (5) the tumor microenvironment (TME) (Fig. 3).

## SEs regulate proliferation

Sustaining proliferation is a hallmark of cancer. While cells in the initial tumor mass are proliferative, disseminating cells in the circulation or those undergoing EMT often interrupt proliferation [82–84]. However, once metastatic cells colonize a secondary site, they are capable of resuming proliferation, even after extended periods of dormancy [84]. Increasing evidence reveals that SE-derived genes play an important role in tumor growth. In human hepatoma cells, KLF6 functions as an oncogene responsible for tumorigenesis. Deleting the KLF6 SE element leads to the upregulation of miR-1301 expression, which in turn enhances p21 and p53 levels to suppress cellular proliferation [85]. In ERα-positive breast cancer, researchers have reported that BRD4 triggers abundant transcription of target genes including *RET* by occupying ERα-controlled SEs. RET then activates signaling cascades to phosphorylate ERα, forming an SE-controlled positive feedback loop that facilitates breast cancer proliferation [70]. Analogously, Cui and colleagues demonstrated that CRISPR/Cas9-mediated deletion of the EphA2-SE effectively downregulates its target gene, *EphA2*, which in turn significantly suppresses cell proliferation and metastasis by inhibiting the PI3K/AKT and Wnt/β-catenin signaling pathways in multiple tumor cells [86].

In addition to SE-derived genes, aberrant ncRNA transcription driven by SEs always contributes to unlimited proliferation. Tissue-specific lncRNA CCAT1 is regulated by SEs in squamous cell carcinoma (SCC). Master TF SOX2 and TP63 co-occupy the CCAT1 promoter and SE regions to activate CCAT1 transcription. Further, CCAT1 forms complexes with SOX2 and TP63 that bind to EGFR SE domains, increasing EGFR expression to activate signaling pathways and SCC proliferation [87]. Similarly, CCAT1-L, the long isoform of CCAT1, is also driven by SEs in CRC. This lncRNA promotes proliferation by driving MYC transcriptional output and enhancing long-range chromatin looping [88]. Further, Zhang et al. found that the oncogenic lncRNA LINC01977 induced by SEs dramatically promotes proliferation and invasion in lung adenocarcinoma (LUAD). LINC01977 is implicated in the activation of the TGF-β/SMAD3 signaling pathway by interacting with SMAD3 in the nucleus to generate the SMAD3/CBP/P300 transcriptional complex, thereby upregulating the expression of downstream ZEB1 [89]. Collectively, these studies affirm the crucial role of SEs in cell proliferation, an indispensable process for cancer metastasis.

## SEs regulate migration and invasion

Migration and invasion are indispensable processes for cancer metastasis, particularly during hematological dissemination [90, 91]. After infiltrating the capillaries within the tumor, cancer cells gain access to the lymphatic or blood vessels, enabling distal metastasis to organs. SEs play a significant role in facilitating metastasis by enhancing cancer cell migration and invasion abilities. For example, a genomic and transcriptomic assessment conducted on 73 CRC tumor tissues and their adjacent normal tissues revealed the presence of 334 gained and 121 lost variant super-enhancer loci (VSELs), including genes linked to well-established oncogenic targets, such as *MYC*, *LIF*, and *VEGFA*. To validate the functional significance of these identified VSELs, the dCas9-KRAB system was employed to suppress enhancer activity, leading to a noteworthy decrease in H3K27ac levels at enhancer loci, reduced expression of associated genes, and diminished capabilities of CRC cells in terms of migration and invasion [92]. Further, Ying et al. reported that the overexpression and abnormal activation of *HOXB8*, a well-known oncogene, were modulated by the SE element rather than by genetic alterations. Inhibition of SE activity resulted in diminished HOXB8 transcription, which subsequently led to a decrease in cell migration and invasion, highlighting the critical importance of SEs in the advancement of cancer [93]. In SCC, a lncRNA named LINC01503 was found to have high expression governed by the SE. Overexpression of LINC01503 was found to enhance cellular proliferation, colony formation, migration, and invasion [94]. Similarly, Linc00152 was found to be upregulated across multiple cancer types and its role in enhancing cell proliferation, migration, and invasion has been confirmed in breast cancer, gastric cancer and kidney cancer [95]. In addition to oncogenes, SE-controlled gene can also function as a tumor suppressor. A recent study indicated that a long SE-regulated gene, *RCAN1.4*, acts as a potential tumor suppressor in breast cancer. Deletion of RCAN1.4-SE with the CRISPR-Cas9 system enhanced migration and invasion abilities, thereby inducing a malignant phenotype in MDA-MB-231 and BT549 cells [96]. The modulation of both oncogenes and tumor suppressor genes by SEs underscores their pivotal roles in the dynamic regulation of cancer progression, particularly in regulating the migration and invasion capabilities of cancer cells.

## SEs regulate stemness

Metastasis is propelled by a subset of cells known as CSCs, which represent only certain tumor cells endowed with multilineage differentiation and self-renewal capacity [4, 97]. SEs are broadly recognized as being important in orchestrating genes that govern cellular states and identity in normal ESCs. In addition, compelling evidence now indicates that cancer cells hijack this mechanism to establish SEs at oncogenes that are critical for CSC regulation [14, 42, 98]. For example, research by Caslini et al. demonstrated that HDAC7 enhances the transcription of several CSC-associated oncogenes, including *C-MYC*, *SLUG*, *CD44*, and *SMAD3*, by elevating H3K27ac levels near the SEs of these genes. Conversely, HDAC7 attenuation was found to suppress the CSC phenotype by downregulating these SE-associated oncogenes [99]. In another context, SEs have been identified as key regulatory elements in augmenting the expression of genes that regulate cancer stemness, leading to increasing the tumorigenic and metastatic potential of human head and neck squamous cell carcinoma (HNSCC). At the mechanistic level, BRD4, NF-κB p65, and Mediator complexes collaborate to form SEs at regions of stemness-associated genes, such as *TP63*, *FOSL1*, and *MET*. Disruptive interventions targeting SEs, such as bromodomain and extra terminal domain inhibitors (BETis) or the CRISPR interference strategy, effectively inhibit CSC self-renewal and ablate CSC populations, ultimately hindering invasive growth and lymph node metastasis in HNSCC [18]. Among patients with breast cancer, racial disparities significantly influence risk factors [100]. African American women show a higher predisposition for developing aggressive, metastatic breast cancer than Caucasian American women. This racial disparity has been attributed to the elevated expression of SOS1, a critical Ras pathway component, in African American patients. Mechanistically, the upregulation of SOS1, mediated by SE regulation, triggers the overexpression of *PTTG1*, a gene associated with c-Met, thus facilitating enhanced colonization of tumor cells in the lungs [101, 102]. . These findings broaden our understanding of SEs in the regulation of stemness and may pave the way for the discovery of novel therapeutic targets.

## SEs regulate EMT

Although substantial research has been conducted on metastasis, the intricate cellular and molecular mechanisms underlying this complex, coordinated process remain largely elusive. One putative mechanism implicates EMT, whereby epithelial cells lose polarity and cell–cell adhesions, enabling cancer cells to have increased mobility, invasion, and stem cell properties to generate new metastatic foci [1, 4]. EMT regulation involves multiple signaling cascades, including TGF, Wnt/β-catenin, and PI3K/AKT pathways [103]. Compelling evidence indicates that aberrant SE activation profoundly impacts epithelial plasticity by modulating these pathways [54, 104, 105]. In pancreatic cancer, *TGFBR2*, which encodes one type of TGF-β receptor, is an SE-associated gene. Deletion of SE regulatory element reduces TGFBR2 expression, consequently impairing EMT and migration [106]. Analogously, AJUBA, an oncogenic protein regulated by SE and linked to an unfavorable prognosis, engages TRAF6 to facilitate the activation of AKT signaling in hepatocellular carcinoma (HCC) [107]. Once activated, AKT signaling contributes to the stability and nuclear translocation of Snail, a key EMT regulator, to increase HCC cell invasiveness and metastatic colonization [108, 109].

In addition to several signaling pathways, core TFs governed by SE signaling regulatory networks, including Zeb, Snail, and YY1, are also involved in EMT regulation [52, 54]. For example, FOSL1, a master TF, has been found to primarily increase tumorigenicity and metastasis in HNSCC by promoting EMT and stemness. Mechanistically, the ncRNA CYTOR enhances the malignant phenotypes of HNSCC cells by promoting formation of phase-separated condensates of FOSL1, leading to the establishment of FOSL1-dependent SEs at a cohort of EMT regulator and pro-metastatic genes, such as *CD44*, *SNAI2*, and *FOSL1* itself [13, 110]. Moreover, the activation of FOSL1 has been observed to occur via stimulation by the MAPK/ERK pathway, a downstream effector of CD44 [111], suggesting an autofeedback loop where FOSL1 acts as the central core. Analogously, *HCCL5*, a novel SE-driven lncRNA, is upregulated and positively correlated with worse overall survival of patients with HCC. Upon TGF-β1 stimulation, ZEB1 directly occupies the promoter and SE region of *HCCL5* to activate its transcription. Consequently, HCCL5 increases the expression of Snail, Slug, ZEB1, and Twist1, resulting in the acceleration of the EMT phenotype to promote the invasion and metastasis of HCC cells [52]. Collectively, these findings delineate the functional and mechanistic interconnectivity between SEs and tumor EMT in driving aggressive carcinomas.

## SEs regulate the TME

**Fig. 4:**
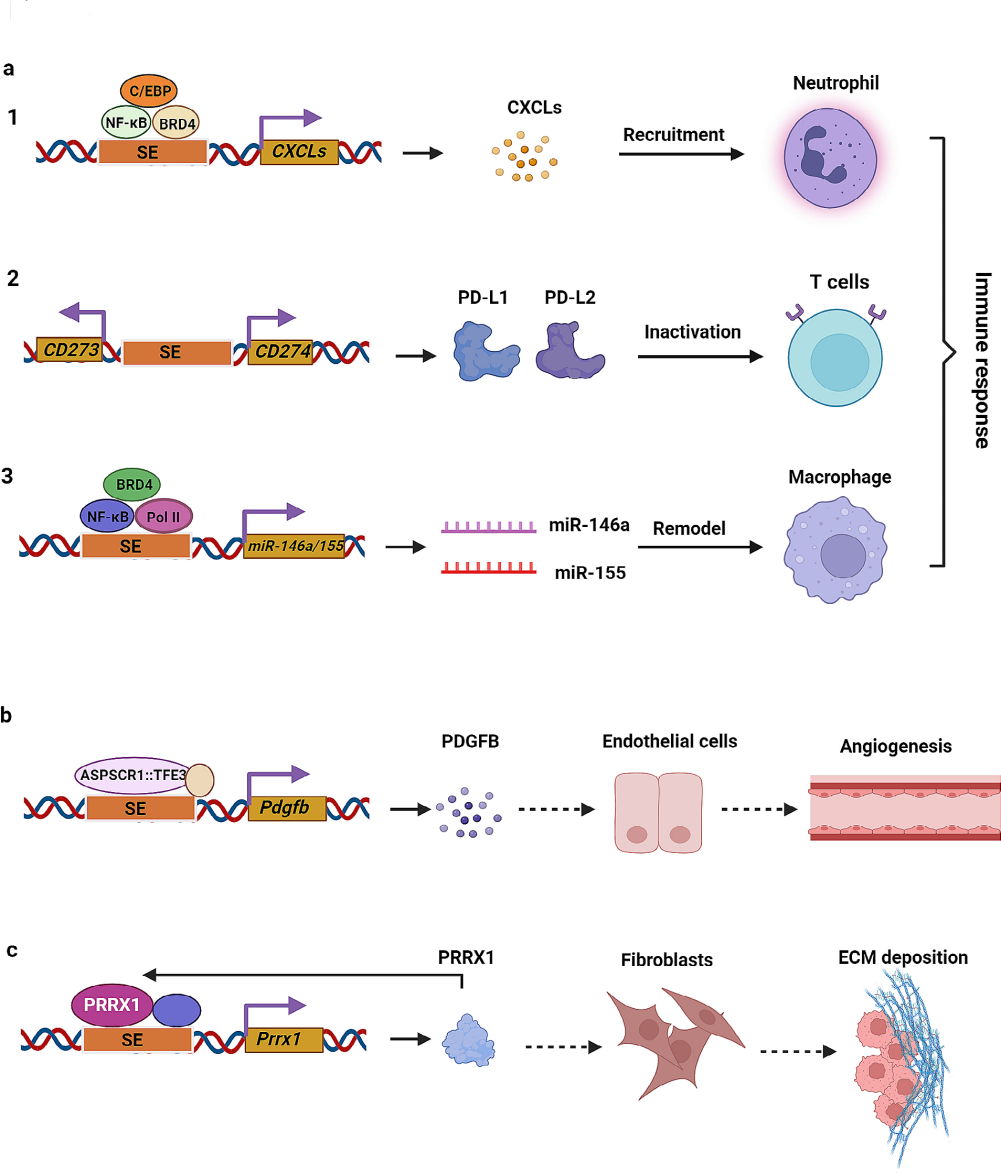
SEs regulate the tumor microenvironment. **a** SEs participate in immune response. **a**1 The transcription factor NF-κB, co-factor BRD4, and C/EBP can bind to the SE regions of multiple CXC chemokines to promote their transcription, thereby priming of neutrophils by inflammatory clear cell renal cell carcinoma (ccRCC) cells to facilitate lung metastasis; **a**2 A SE located between the *CD274* and *CD273* genes drives the expression of PD-L1 and PD-L2, leading to immune evasion and resistance to T cell-mediated killing; **a**3 NF-κB, BRD4, and RNA Pol II bind to the SE region of miR-146a and miR-155 to enhance their transcription. These microRNAs can be transported to tumor-associated macrophages through exosomes, stimulating M2-like macrophage polarization and promoting cancer progression. **b** ASPSCR1::TFE3, the fusion TF, orchestrates the angiogenic program to promote the development of cancer through enhancing SE activity at critical genes, such as *Pdgfb*, *Rab27a*, *Sytl2*, and *Vwf*. **c** SE orchestrates the transcription of *PRRX1* gene, encoding a critical TF. The PRRX1 protein subsequently engages in autoregulatory interaction with its corresponding SE, thereby forming a critical regulatory loop, which is essential for initiating the transition of fibroblasts into a myofibroblastic phenotype and enhancing the deposition of the extracellular matrix

The TME is extremely complex and heterogeneous, comprising a conglomeration of soluble factors, ECM, immune cells, cancer-associated fibroblasts (CAFs), endothelial cells, and pericyte cells [112, 113]. The TME is implicated in the induction of proliferation, ECM deposition, angiogenesis, evasion of immune surveillance, and immune system suppression [114–116]. The bidirectional interactions between tumor cells and the TME are crucial in adapting to new conditions and facilitating cancer initiation, progression, and metastasis. A plethora of studies have suggested that SEs can directly or indirectly change the communication between cells and their TME, ultimately promoting cancer angiogenesis, ECM remodeling, and immune invasion, which are key factors in cancer metastasis (Fig. 4) [81, 117, 118].

### SEs participate in the immune response

The TME is profoundly impacted by the ability of cancer cells to manipulate anti-tumor immunity through an array of mechanisms. These include the secretion of immunomodulatory cytokines and chemokines, termed cancer cell-intrinsic inflammation, which enables tumors to evade immune surveillance, a major obstacle facing effective cancer immunotherapy [119, 120]. The programmed cell death protein 1 (PD-1)/programmed death ligand 1 (PD-L1) immune checkpoint pathway represents one such mechanism exploitable by tumors to circumvent T cell-mediated killing [121]. Emerging evidence reveals that SEs play a pivotal role in shaping anti-tumor immune responses by modulating inflammation and immune evasion (Fig. 4a).

During the advanced progression of clear cell renal cell carcinoma (ccRCC), systemic inflammation commonly occurs and can trigger neutrophil-dependent lung metastasis. Research indicates a significant role for SEs in the upregulation of a suite of inflammation-associated genes. The employment of a pharmacological inhibitor of BRD4, JQ1, has been shown to downregulate the transcription of numerous CXC motif chemokines, restrain neutrophil activity, and obstruct the progression of neutrophil-mediated lung metastasis [117]. Similarly, in breast cancer, robust expression of PD-L1 and PD-L2 is orchestrated by a SE termed PD-L1L2-SE. Genetic ablation of PD-L1L2-SE results in reduced expression levels of PD-L1 and PD-L2, consequently undermining tumor cells’ capability for immune evasion and enhancing their susceptibility to cytotoxic T lymphocytes [122]. Intriguingly, a recent study reported that lactate can increase the percentage of stem-like TCF-1-expressing CD8^+^ T cells and augment anti-tumor immunity. Mechanistically, lactate inhibits histone deacetylase activity, leading to enhanced H3K27 acetylation at the *Tcf7* SE locus and increasing its expression [123]. Further, ChIP-seq analyses performed by Duan and colleagues revealed that the transcription factors NF-κB and BRD4, along with RNA Pol II, directly engage with the SE region of miR-146a and miR-155, facilitating their upregulated transcription [124]. Both miR-146a and miR-155 are microRNAs implicated in inflammatory processes. These miRNAs can be secreted by cancer cells into the TME and then incorporated into tumor-associated macrophages via exosomal transfer, promoting an immunosuppressive, M2-like macrophage phenotype that supports tumor progression [125, 126]. These findings suggest that modulating SE activity can potentiate the responsiveness of tumor cells to immunotherapeutic strategies by restructuring the tumor immune milieu.

### Involvement of SEs in angiogenesis

Angiogenesis, defined as the genesis of novel vasculature from pre-existing vessels, is essential for neoplastic proliferation and metastasis because it delivers necessary nutrients and energy to the tumor [127]. This dynamic and intricate process is governed by a variety of molecules that are implicated in regulating endothelial cell proliferation and migration [128]. Recently, scientists have identified key oncogenic SEs that regulate cancer angiogenesis by boosting expression of crucial molecules or activating signaling pathways, shedding light on the complex interplay between neoplastic cells and their surrounding stroma.

In alveolar soft part sarcoma (ASPS), expansive vascularity and marked metastatic potential are cardinal features, recently attributed to orchestration of angiogenic genetic programs by the distinctive ASPSCR1-TFE3 fusion oncoprotein [129, 130]. This aberrant TF remodels the SE landscape to stimulate the expression of pro-angiogenic mediators such as PDGFB, Rab27a, Sytl2, and VWF. Depletion of ASPSCR1-TFE3 or JQ1 treatment reduces SE activity at these loci, leading to the disruption of the vascular network and attenuation of ASPS advancement (Fig. 4b) [131]. Further insights from Nguyen et al. found that the acetyltransferases CBP/p300-mediated acetylation at lysine 13 of HOXB13 acts as a prognostic indicator in metastatic castration-resistant prostate cancer (CRPC). This acetylation fosters the formation of tumor-specific SEs at the loci of genes that boost angiogenesis (e.g. VEGFA, angiopoietins), facilitating tumor growth and vascular development [132]. Additionally, angiotensin II (Ang II) has been proposed as a significant contributor to angiogenesis and metastasis in multiple carcinomas, including ovarian cancer [133], breast cancer [134], and HCC [135]. Das et al. reported that Ang II modulates enhancers/SE repertoires to drive associated gene transcription, resulting in vascular smooth muscle cell proliferation and angiogenesis [136]. Collectively, these findings highlight that harnessing SE dynamics represents a promising approach to restrict pathological angiogenesis and resultant metastatic dissemination across cancer subtypes.

### SEs are implicated in ECM remodeling

Accumulating evidence shows that the ECM, a critical component of the TME, plays a crucial role in cancer metastasis [137]. Various remodeling mechanisms lead to alterations in the ECM, which can generate a cancer-supporting matrix that contributes to the tumor’s pathology [138]. SEs can directly or indirectly impact ECM remodeling by governing the transcription of ECM components and associated regulators.

Recently, approximately 80 SE-associated lncRNAs have been identified in hepatic stellate cells (HSCs), which are responsible for regulating ECM stiffness [139]. A strong interaction exists between ECM stiffness and HSC activation, forming a positive feedback loop in the progression of cancer metastasis [140, 141]. Similarly, Wang et al. revealed that SEs promote the expression of various genes involved in pancreatic stellate cell activation. This results in abundant ECM deposition and cytokine secretion, impeding drug access to tumor tissues [142]. Further, SE-associated genes have also been identified as CAFs. Investigations have revealed that PRRX1, a key TF implicated in ECM stiffness, exhibits elevated expression driven by SE activation in CAFs. Intriguingly, PRRX1 can engage directly with its own SEs and other SEs, creating a central regulatory network that promotes tumorigenesis, metastasis, and cancer recurrence (Fig. 4c) [143]. More importantly, the pro-inflammatory cytokines IL-6 and IL-8, which are secreted by CAFs, have been found to induce BRD4 expression in CRC cells. This leads to BRD4 and STAT3 interaction, enhancing SE activity and promoting more robust oncogenic transcription, ultimately contributing to BET inhibitor resistance [144]. Altogether, targeting SE dynamics in HSCs or CAFs represents a promising therapeutic strategy to disrupt the tumor-supportive ECM and mitigate metastasis.

## Pharmacological targeting SE-driven transcriptional program in cancer metastasis

**Fig. 5:**
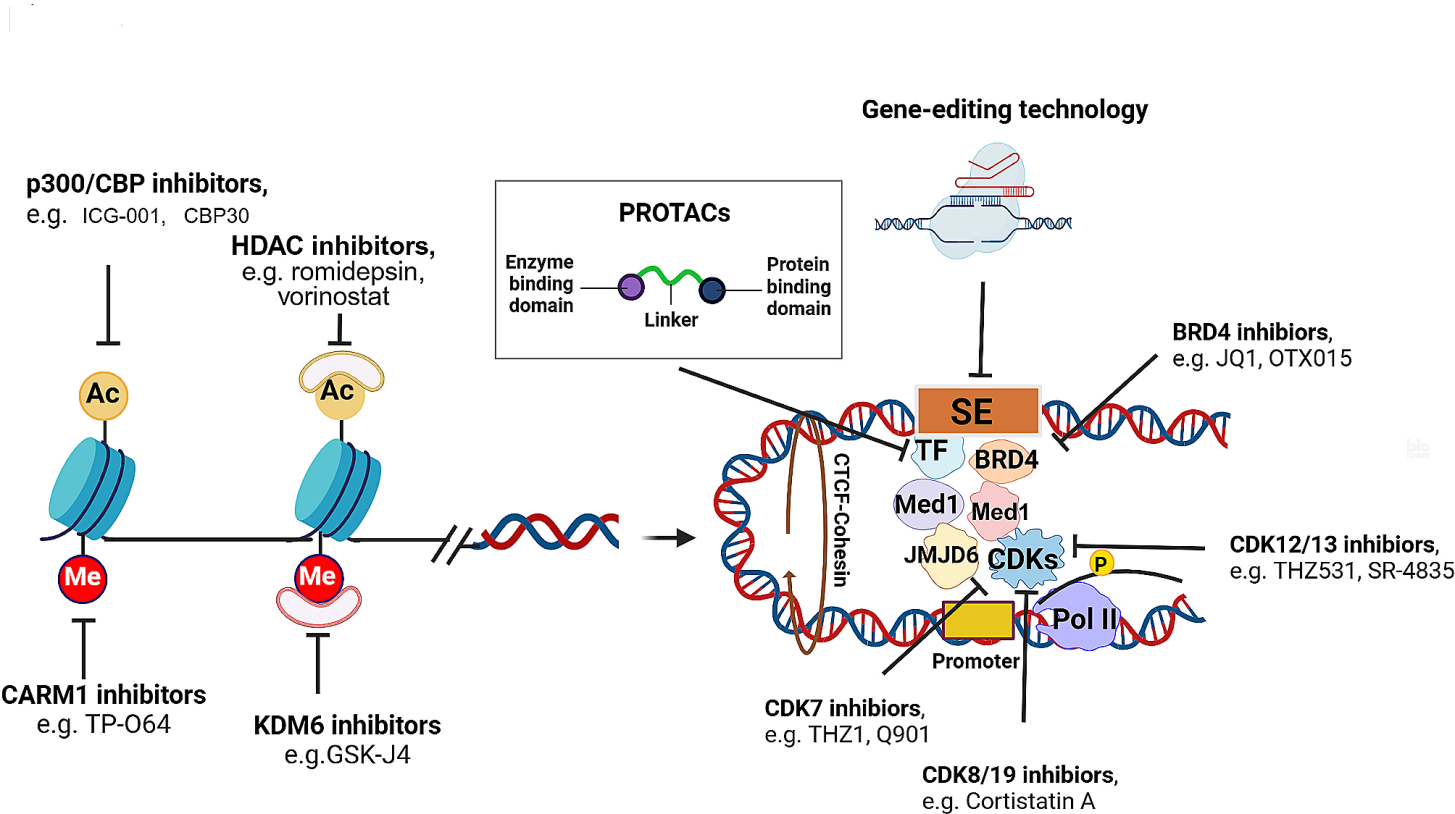
Pharmacological targeting SE-driven transcriptional program in cancer. Inhibition of histone deacetylase enzymes (HDACs) with romidepsin or virinostat disrupts acetylation marks levels, impeding the interactions between SEs and promoters. Suppression of the enzymatic activity of histone acetyltransferases CBP/p300 with ICG-001 or CBP30 perturbs SE formation. Treatment with coactivator-associated arginine methyltransferase 1 (CARM1) inhibitor TP-064 hampers the methylation of BAF155, impairing the recruitment of BRD4 and the formation of SEs. Inhibition of the H3K27 demethylase KDM6 with GSK-J4 leads to widespread enhancer reorganization, particularly affecting stemness genes regulated by SEs. JQ1 and OTX015 specifically target BRD4, leading to a reduction in the recruitment of Mediator, BRD4, and RNA Pol II at SE sites. Inhibitors targeting CDKs, upregulate or downregulate the transcription of SE-associated genes through affecting phosphorylation C-terminal domain (CTD) of RNA Pol II. Proteolysis-targeting chimeras (PROTACs) can selectively hijack BRD4, CDKs and TFs into the ubiquitin-proteasome system to elicit its degradation, resulting to interruption of SE-driven transcriptional program. CRISPR/Cas9-mediated genetic perturbation can directly targeting individual components within SEs

Recent research has indicated that tumor cells exhibit a greater overall transcription output than normal cells, increasing the opportunities to engage oncogenic pathways. Therefore, targeting the formation and activation of SE-driven oncogene transcription may present a promising approach to cancer treatment. Regarding the various protein constituents within the regulatory pathway, different strategies for disrupting SEs are categorized into the following types: (1) BET inhibitors, (2) CDK inhibitors, (3) targeting epigenetic modulators, (4) targeting SE remodeling, and (5) gene-editing technology (Fig. 5). Among them, several SE antagonists are presently under clinical evaluation, with the aim to determine their therapeutic efficacy and safety profile in the management of malignancies (Table 3).

**Table 3:** SE-associated therapeutic agents for clinical trials in cancers

Target	Therapeutic agents	Tumor type	Clinical trial	Status
BET/BRD4 domain	BMS-986,158/BMS-986,378	Pediatric brain tumors	NCT03936465	Phase 1
	ZEN003694	Prostate cancer	NCT02705469	Phase 1
	ABBV-075	Advanced solid tumors	NCT02391480	Phase 1
	OTX015	Hematologic malignancies	NCT01713582	Phase 1
	RO6870810	Multiple myeloma	NCT03068351	Phase 1
	CC-90,010	Advanced solid tumors and relapsed NHL	NCT03220347	Phase 1
CDK7	CPI-0610	Progressive lymphoma	NCT01949883	Phase 1
	PLX51107	Myelodysplastic syndrome or AML	NCT04022785	Phase 1
	SF1126	Advanced hepatocellular carcinoma	NCT03059147	Phase 1
	INCB054329	Advanced malignancies	NCT02431260	Phase 1/2
	Q901	Advanced solid tumors	NCT05394103	Phase 1/2
	SY 5609		NCT04247126	Phase 1
	SY-1365		NCT03134638	Phase 1
	XL102		NCT04726332	Phase 1
	CT7001		NCT03363893	Phase 1/2
	LY3405105		NCT03770494	Phase 1/2
CDK8	RVU120 (SEL120)	AML	NCT04021368	Phase 1
CDK9	AZD4573	AML	NCT03263637	Phase 1
	VIP152	Relapsed/Refractory CML	NCT04978779	Phase 1
	BAY 1,251,152	Advanced blood cancers	NCT02745743	Phase 1
		Solid tumors and aggressive NHL	NCT02635672	Phase 1
	GFH009	Hematologic malignancies	NCT04588922	Phase 1/2
	KB-0742	Relapsed/refractory solid tumors or NHL	NCT04718675	Phase 1/2
	PRT2527	Hematologic malignancies	NCT05665530	Phase 1
		Advanced solid tumors	NCT05159518	Phase 1
	BAY1143572	Acute leukemia	NCT02345382	Phase 1
		Advanced cancer	NCT01938638	Phase 1
CBP	ICG-001 analog	Advanced solid tumors	NCT01764477	Phase 1
		Advanced myeloid malignancies	NCT01606579	Phase 1/2
		Advanced or metastatic pancreatic adenocarcinoma	NCT01764477	Phase 1
		Metastatic CRC	NCT02413853	Phase 2
HDAC1/2	Romidepsin	Recurrent or Metastatic TNBC	NCT02393794	Phase 1/2
STAT3	KT-333	NHL, PTCL, CTCL, LGL-L, solid tumors	NCT05225584	Phase 1
AR	ARV-110	mCRPC	NCT03888612	Phase 1/2
ER	ARV-471	ER^+^/HER2^−^advanced or metastatic breast cancer	NCT05463952	Phase 1
CRISPR-Cas9	Autologous CRISPR-Cas9 modified hematopoietic stem cells	β-thalassemia or sickle cell disease	NCT04208529	Phase 3

AML: acute myeloid leukemia; CML: chronic lymphocytic leukemia; CRC: colorectal cancer; CTCL, cutaneous T-cell lymphoma; LGL-L, large granular lymphocytic leukemia; mCRPC, metastatic castration-resistant prostate cancer; NHL: Non-Hodgkin’s Lymphoma; PTCL, peripheral T-cell lymphoma; TNBC: triple-negative breast cancer

## BET inhibitors

The members of the BET protein family, including BRD1-4 and BRDT, are capable of recognizing histone proteins through binding to acetylated lysine residues, acting as readers in epigenetic regulation [145]. Strategic targeting of BET family proteins, particularly BRD4, leads to a significant decrease in the occupancy at SE sites, which results in the transcriptional repression of oncogenes, eliciting anti-tumorigenic effects both in vitro and in vivo [146].

Inhibitors of BET proteins can be categorized as either monovalent, including JQ1, CPI-0610, GSK525762, and OTX015, or bivalent, represented by AZD5153 and MT1 [147, 148]. Of these, JQ1 is the most extensively studied small-molecule BET inhibitor, which competes with BRD4 for binding to chromatin, displacing Mediator and RNA Pol II from enhancer constituents. Administration of JQ1 has been demonstrated to disrupt SE integrity, eliciting oncogene transcriptional repression and subsequently inhibiting growth and metastasis in both hematological malignancies and solid tumors [149–151]. Several BET inhibitors have been brought into phase I or II clinical trials. However, despite remarkably encouraging preclinical evidence, single agent efficacy in humans has proven transient and infrequent. Resistance mechanisms have also emerged, exemplified in acute myeloid leukemia (AML) by increased Wnt/β-catenin signaling within CSCs. Blocking Wnt/β-catenin restored sensitivity to JQ1 both in vitro and in vivo [152]. A recent study revealed that BRD4 resistance can arise from mechanisms other than Wnt/β-catenin activity alone. In CRC cells, proinflammatory factors secreted by CAFs trigger the activation of JAK2, resulting in the phosphorylation of BRD4 at tyrosine 97/98. This phosphorylation stabilizes BRD4, enhancing its chromatin-binding capacity and reducing its sensitivity to BET inhibitors, ultimately leading to resistance [144]. These findings underscore the potential utility of strategically combining BET inhibition with complementary approaches for enhanced anticancer efficacy.

In addition to the small-molecule inhibitors that aim to occupy the bromodomain pocket of BRD4, an emerging class of chimeric degraders, termed proteolysis-targeting chimeras (PROTACs), can selectively hijack BRD4 into the ubiquitin-proteasome system to elicit its degradation [153]. As bifunctional molecules, PROTACs bridge BRD4 to an E3 ubiquitin ligase through high affinity binding to each protein, stimulating ubiquitination and subsequent proteasomal destruction of the transcriptional co-activator. Rather than simply inhibiting bromodomain activity, PROTAC-mediated BET protein degradation may achieve more complete and sustained therapeutic suppression [154]. Lead BET-directed PROTACs have already exhibited powerful antitumor activity in neuroblastoma [155], leukemia [156], and castration-resistant prostate cancer [157], overcoming the limitations of traditional bromodomain antagonism. For example, ARV-825, a potent PROTAC, engages BRD4 with the E3 ubiquitin ligase cereblon, resulting in rapid, effective, and sustained degradation of BRD4. Mounting evidence demonstrates that ARV-825 treatment leads to sustained BRD4 degradation, effectively reducing the levels of oncogenic proteins such as c-MYC, PIM1, and CXCR4. This ultimately results in impaired proliferation, apoptosis induction, and reversal of stroma-mediated drug resistance in AML and Burkitt’s lymphoma [158–161]. Additional heterobifunctional small molecule degraders of BET bromodomain proteins, including dBET57 [162], dBET1 [163], dBET6 [156], and ARV-771 [157], have been developed using PROTAC technology. Despite the nascent stage of translation, PROTAC-mediated BET degradation constitutes a promising therapeutic approach for clinical advancement.

## Cyclin-dependent kinase inhibitors

Cyclin-dependent kinases (CDKs) constitute a family of serine/threonine protein kinases that regulate key cellular processes including cell cycle progression (CDK1, CDK2, CDK4, CDK6) and transcriptional control (CDK7, CDK8, CDK9, CDK12, CDK13, CDK19) [164–166]. CDK7 forms an integral component of TF IIH (TFIIH), mediating the phosphorylation of RNA Pol II C-terminal domain (CTD) heptapeptide repeats at serine 5 (Ser5) and serine 7 (Ser7) residues to initiate transcription [167]. In addition, CDK7 phosphates to CDK9, a part of the positive transcription elongation factor b (P-TEFb), which then phosphorylates serine 2 (Ser2) residues within the RNA Pol II CTD to promote transcriptional elongation [168]. The highly homologous kinases CDK8 and CDK19 associate with Mediator complexes to regulate transcription, in part by targeting and inhibiting CDK7 activity [169, 170]. Beyond CDK7/9, CDK12, and CDK13 can also promote the phosphorylation of RNA Pol II CTD at Ser2, Ser5 and Ser7, leading to transcriptional initiation and elongation [171].

Research has demonstrated that the activation of transcription by SEs is closely linked to the recruitment of CDK7-containing TFIIH, CDK9-containing p-TEFb, CDK8/CDK19, and CDK12/13. Therefore, inhibitors that specifically target CDKs can diminish SE activity, thereby hindering the onset and advancement of cancer as well as reversing chemoresistance. THZ1, a small-molecule inhibitor that obstructs the phosphorylation of Ser2, Ser5 and Ser7 by selectively targeting CDK7, shows the capacity to block SE-driven transcription at low concentrations and repress general transcription at high doses [172]. The effectiveness of CDK7 inhibition has been demonstrated in various aggressive cancer types, including small-cell lung cancer [20], triple-negative breast cancer (TNBC) [21], MYCN-amplified neuroblastoma [47], osteosarcoma [173] and pancreatic ductal adenocarcinoma [174]. Similarly, CDK9 has been implicated in the SE-driven transcription of short-lived proteins critical for oncogenic survival, such as MYC and MCL1 [175]. This dependency on transcription makes cancer cells more vulnerable to CDK9 inhibitors, such as AZD4573, which has demonstrated significant anti-tumor effectiveness in preclinical models of diffuse large B-cell lymphoma [176]. Further, Cortistatin A, a specific inhibitor of CDK8/CDK19, exhibits potent antiproliferative effects against various leukemia cell lines in vitro and shows strong efficacy in AML mouse models via increasing the expression of SE-associated genes with tumor suppressor roles, including *IRF1*, *IRF8*, and *CEBPA* [177]. Moreover, new small-molecule inhibitors of CDK12/13, namely THZ531 and SR4835, substantially reduce the transcription of DNA damage response and critical SE-associated genes, eventually leading to retardation of tumor proliferation and metastasis in T-ALL [178] and CRC cells [8]. Therefore, therapeutic targeting of CDKs that are crucial for SE function represents a promising anticancer strategy warranting further investigation.

### Targeting epigenetic modulators

The posttranslational modification of histones, including acetylation/deacetylation and methylation/demethylation, is instrumental in chromatin architecture and gene transcriptional regulation [179]. Chromatin histone acetylation is regulated by the dynamic balance between histone acetyltransferases (HATs) and histone deacetylase enzymes (HDACs) [180]. Acetylated histones destabilize nucleosomes, leading to enhanced chromatin accessibility for TFs. Further, histone methyltransferases (HMTs) and demethylases (HDMs) catalyze methylation, either activating or repressing transcription depending on residue positioning [181]. High-density H3K27ac or H3M4me1 is a marker to identify SEs, permitting transcriptional induction of downstream targets in response to stimuli.

Emerging evidence reveals that epigenetic regulators are implicated in SE-dependent transcriptional programs, significantly influencing the development and progression of cancer [180, 182]. Frequent loss-of-function mutation of the H3K4 methyltransferase KMT2D has been observed in lung tumors, where it acts as a suppressor of malignant growth [183]. Deficiency of KMT2D leads to abnormal metabolic reprogramming by impairing SE activity of *PER2*, a tumor suppressor gene, increasing the sensitivity of lung cancer cells to glycolytic inhibitors [184]. Similarly, Kim and colleagues found that coactivator-associated arginine methyltransferase 1 (CARM1) promotes the arginine-methylation of BAF155 to drive TNBC metastasis partially through activating SE-addicted oncogenes via the recruitment of BRD4 [80]. In addition, loss of KDM6A, a demethylase that targets H3K27me3, has been involved in the initiation and the development of squamous-like, metastatic pancreatic cancer in females by disrupting the COMPASS-like complex and abnormally activating SEs that regulate the oncogenes *ΔNp63*, *MYC*, and *RUNX3* [185]. On the other hand, targeted inhibition of KDM6 with the small molecule GSK-J4 has been shown to profoundly halt CRC expansion by eliminating tumor-propagating cells. Mechanistically, GSK-J4 reshapes the SE landscape at critical stemness genes, *ID1* [186].

Apart from targeting HMTs or HDMs, inhibition of histone acetyltransferases (HATs), such as p300/CBP, using with specific compounds (e.g. ICG-001 or CBP30) also perturbs SE formation, thereby attenuating progression in LUAD [45] and pediatric high-grade gliomaspheres [187]. Fascinatingly, HDACs, enzymes that remove acetyl groups, similarly control SE activity in a counterintuitive fashion. Key members of this enzyme family, including HDAC1, HDAC2, and HDAC7, play pivotal roles in maintaining H3K27ac markers, thereby facilitating the activation of SE-regulated oncogenes within cancer cells. The inhibition of HDACs with romidepsin or vorinostat leads to a decrease in H3K27ac levels at SE sites, thereby reducing transcriptional activation and yielding promising anti-tumor activity in preclinical models of breast cancer [99] and glioblastoma models [188]. Although unexpected given their opposing biochemical activities, HDACs are critical for maintaining appropriate acetylation levels, as extreme hyperacetylation can also disrupt SE-dependent gene expression. In summary, we aim to highlight the involvement of diverse histone modifiers in SE regulation relevant to cancer and underline the therapeutic potential of targeting these enzymes to hinder cancer progression.

## Targeting SE reprograming

Transcriptional dysregulation is a prevalent characteristic of human disease, especially in cancers [10, 189]. In normal cells, gene expression programs are precisely regulated. However, tumor cells rely heavily on aberrant gene transcriptional programs [190, 191]. Many oncogenic drivers are substantially overexpressed, which contributes to the activation of crucial oncogenic signaling pathways and promotes metastasis [140, 192]. Recent research suggests that SE reprogramming serves as the key factor driving alterations in the transcriptional programs of oncogenes in malignancies [9, 140].

The initiation of SE reprogramming primarily involves the binding of pioneer TFs, which leads to alterations in the regulatory landscapes of hundreds of putative SEs [193–195]. In eukaryotes, nucleosomes are wrapped in “closed” or “silent” chromatin, preventing TFs from binding and the transcriptional machinery from starting [196]. Pioneer TFs are TFs that possess the unique ability to open the “closed” chromatin to alter cell fates through the recognition and binding of DNA target sequences within the enhancer or SE region. Subsequently, co-activator proteins such as histone modifications, Mediator complexes, and chromatin remodelers are introduced to enhancers or SEs to active transcriptional programs [196, 197]. In recent years, the investigation of pioneer factors has highlighted their important roles in SE remodeling during tumor progression. For example, FoxA1, the first identified pioneer TF, has been shown to interact with a target DNA sequence wrapped around nucleosomes to open chromatin, which results in enhancer reprogramming and subsequent tumorigenesis in prostate [198], breast [199], and pancreatic carcinoma [7]. Further, a comparison between SCC and paired healthy counterparts revealed that H3K27ac occupancy at chromatin is dramatically changed, suggesting that SE remodeling occurred in SCC. Mechanistically, ΔNp63α, a prominent isoform of TP63, functions as a pioneer factor, binding closed chromatin to increase chromatin accessibility at enhancer sites through interacting with epigenetic modulators and TFs, including KLF4 and p53 [194]. In addition, the process of CRC hepatic metastasis has been associated with large-scale reorganization of the TE and SE landscapes, facilitated by tissue-specific pioneer factors FOXA2 and HNF1A that promote liver-specific gene expression [9].

Given the power of pioneer TFs to reprogram the epigenome and determine cell identity, it is not surprising that their misexpression presents an “Achilles heel” in cancer. Nevertheless, due to structural heterogeneity and the absence of accessible active sites, these proteins have been deemed undruggable, resulting in only a handful of them being effectively targeted by conventional small molecules [200]. Over the past few years, novel small molecule-based PROTACs alongside strategies using DNA motifs recognized by TFs to guide PROTAC action have shown superior efficacy in metastatic cancers, offering a promising avenue for targeting undruggable TFs and potentially providing therapeutic benefits [201]. To date, several PROTACs targeting TFs have advanced into the clinical. The STAT3 degrader KT-333 is currently in Phase I clinical trials [202]. The androgen receptor (AR) degrader, ARV-110, has demonstrated therapeutic responses in prostate cancer patients with both wild-type AR and those harboring AR T878A and H875Y mutations [203]. Additionally, the ER degrader ARV-471 has shown promise in treating breast cancer patients with both wild-type and mutant ER [204]. These small-molecule-based PROTACs, characterized by improved oral bioavailability, low toxicity, and superior efficacy, provide therapeutic advantages over traditional methods of inhibiting transcriptional activity.

### Targeting SEs by gene editing

Recently, the use of the CRISPR/Cas9 system in cancer research and treatment has increased, yielding remarkable outcomes [205, 206]. Due to its high efficiency and precision, the CRISPR/Cas9 technique may become the most direct and precise way to modulate SE activity. Multiple preclinical models have provided evidence supporting the feasibility of employing genetic approaches to target SEs. For example, in mutant RUNX1-expressed AML cells, deleting the RUNX1 enhancer within its intragenic SE using CRISPR/Cas9 leads to the suppressed expression of RUNX1, subsequently decelerating cell growth and increasing cell death [207]. Similarly, CRISPR-Cas9-mediated deletion of individual components of the *TP63* SEs reduces its expression levels and restrains the proliferation of esophageal squamous cell carcinoma (ESCC) cells, unveiling the oncogenesis role of TP63 in tumor malignancy [208]. Further, the CRISPR/Cas9 technique has been employed in a treatment that involves modifying the erythroid-specific SE at *BCL11A* gene in hematopoietic stem and progenitor cells, followed by autologous transplantation to subjects with sickle cell anemia or transfusion-dependent β-thalassemia (NCT04208529) [67]. A long-term follow-up study is currently underway to assess the efficacy and safety of this treatment.

Despite its efficiency and convenience, CRISPR-Cas9 gene editing has certain limitations, including inefficient base editing and off-target effects, which constrain its application in some cases. Hence, careful evaluation of the therapeutic efficacy and safety of gene-editing technology for clinical use is crucial. Currently, a newly developed precise gene-editing tool called Prime Editor has been introduced. This advanced tool is designed to precisely edit SE formations and address the issues of nonspecific base editing associated with the CRISPR-Cas9 system [209]. Importantly, this modern technology has great potential for providing personalized precision therapy to patients with cancer, considering the specific characteristics of SEs.

## Conclusions

Oncogene addiction renders cancer cells profoundly addicted to the abundant transcriptional output governed by SEs to maintain their malignant state. These tumor dependencies can offer a framework for identifying targets in cancers with unknown drivers or those prone to metastasis. Although there is strong evidence that SEs modulate cell identity genes, the regulatory mechanism of SEs still lacks a clear understanding. Thus, using a comprehensive approach that integrates 5 C with innovative techniques such as Hi-ChIP, Hi-C, ATAC-seq, and single-cell sequencing, in conjunction with an enhanced comprehension of phase separation and the CRISPR genome-editing tool, can reveal the intrinsic 3D structure of SEs and their gene interactions. This strategy will shed light on the mechanisms underlying SE-mediated transcriptional regulation and oncogenesis.

Aberrant activation of SEs plays an important role in promoting metastasis by increasing the proliferation ability, EMT, migration, invasion, CSC formation, and TME modulation. Compelling studies have demonstrated an enrichment of SE-associated genes participating in the metastatic cascade in tumors, such as CRC [9], breast cancer [80], ESCC [210], and pancreatic cancer [7]. Moreover, the SE-regulated overexpression of oncogenes is strongly linked to the poor prognosis, including shorter overall survival and metastasis-free survival in osteosarcoma [211] and hepatocellular carcinoma [212], can facilitate the identification and development of tumor biomarkers. This, in turn, may guide the selection of targeted treatment options for cancer patients, especially those requiring urgent precision medicine interventions. However, these observations are yet to be validated by directly interrogating the functional implications of SE using an in vivo model. Further research should employ genome editing techniques to edit SEs or their regulatory factors in animal models, which will provide definitive evidence regarding the regulation of metastasis by SEs and refine our understanding of the causal influence of SEs on metastatic dissemination. In addition, it is necessary to investigate whether metastatic tumors share common SEs regardless of the tissue of origin or dissemination sites.

Currently, numerous small-molecule inhibitors that target SE-related transcriptional machinery have undergone evaluation in both preclinical models and clinical trials, demonstrating promising efficacy against diverse advanced malignancies. However, resistance to treatments with BETis and CDK7 inhibitors has been observed in many cancer cells [152, 213]. Understanding the alterations of SEs in drug-resistant and relapsed tumor models will be crucial for uncovering the underlying mechanisms and discovering new vulnerabilities. Additionally, it is essential to recognize that targeting SEs for cancer treatment may lead to side effects that cannot be overlooked. Disruption of SEs might concurrently inhibit the transcription of particular tumor suppressor genes that are SE-dependent, potentially resulting in pronounced toxicity and adverse outcomes. Comprehensive research is imperative to thoroughly assess the safety and effectiveness of SE-targeting inhibitors, both as monotherapy or in combination with other antitumor agents.

## Data Availability

No datasets were generated or analysed during the current study.

## Abbreviations

AML: acute myeloid leukemia
Ang II: angiotensin II
AR: androgen receptor
ASPS: alveolar soft part sarcoma
ATAC-seq: accessible chromatin using sequencing
ATLL: adult T-cell leukemia/lymphoma
BETi: bromodomain and extra terminal domain inhibitor
BRD4: Bromodomain-containing protein 4
CAF: cancer-associated fibroblast
CARM1: coactivator-associated arginine methyltransferase 1
ccRCC: clear cell renal cell carcinoma
CDK: Cyclin-dependent kinase
CDK7: cyclin-dependent kinase 7
ChIA-PET: chromatin interaction analysis with paired-end tag
ChIP-seq: chromatin immunoprecipitation sequencing
circRNA: circular RNA
CRC: core regulatory circuitry
CRC: colorectal cancer
CRISPRa: CRISPR activation
CRISPRi: CRISPR inhibition
CRPC: castration-resistant prostate cancer
CSC: cancer stem cell
CTC: circulating tumor cell
CTCF: CCCTC-binding factor
CTD: C-terminal domain
CUT&Tag: Cleavage Under Targets and Tagmentation
EBV: Epstein–Barr virus
ECM: extracellular matrix
EMT: epithelial-mesenchymal transition
eRNA: enhancer RNA
ERα: estrogen receptor alpha
ESC: embryonic stem cell
ESCC: esophageal squamous cell carcinoma
FAIRE-seq: formaldehyde-assisted isolation of regulatory elements sequencing
H3K27ac: histone H3 lysine 27 acetylation
H3K4me1: histone H3 lysine 4 monomethylation
HAT: histone acetyltransferase
HCC: hepatocellular carcinoma
HDAC: histone deacetylase enzyme
HDM: histone demethylase
HiCAR: Hi-C on Accessible Regulatory DNA
HMT: histone methyltransferase
HNSCC: human head and neck squamous cell carcinoma
HOMER: Hypergeometric Optimization of Motif Enrichment
IDR: intrinsically disordered region
LGL-L: large granular lymphocytic leukemia
LLPS: liquid-liquid phase separation
lncRNA: long noncoding RNA
LUAD: lung adenocarcinoma
mCRPC: metastatic castration-resistant prostate cancer
Med1: Mediator Subunit 1
miRNA: microRNA
ncRNA: noncoding RNA
NGS: Next-generation sequencing
NHL: Non-Hodgkin’s Lymphoma
PD-1: protein 1
PD-L1: programmed death ligand 1
PLAC-seq: chromatin immunoprecipitation sequencing
Pol II: Polymerase II
PROTAC: proteolysis-targeting chimera
PTCL: peripheral T-cell lymphoma
P-TEFb: positive transcription elongation factor b
ROSE: Rank Ordering of Super Enhancers
SCC: squamous cell carcinoma
SE: Super-enhancers
Ser2: serine 2
Ser5: serine 5
Ser7: serine 7
SNP: single-nucleotide polymorphism
STARR-seq: self-transcribing active regulatory region sequencing
TAD: Topologically associating domain
T-ALL: T-cell acute lymphoblastic leukemia
TE: typical enhancer
TF: transcription factor
TGF: transforming growth factor
TME: tumor microenvironment
TNBC: triple-negative breast cancer
TSS: transcription start site
VSEL: variant super-enhancer loci

## References

[1] Mittal V (2018). Epithelial mesenchymal transition in Tumor Metastasis. Annu Rev Pathol.

[2] Xie X, Li Y, Lian S, Lu Y, Jia L (2022). Cancer metastasis chemoprevention prevents circulating tumour cells from germination. Signal Transduct Target Ther.

[3] Siegel RL, Miller KD, Wagle NS, Jemal A (2023). Cancer statistics, 2023. CA Cancer J Clin.

[4] Ganesh K, Massagué J (2021). Targeting metastatic cancer. Nat Med.

[5] Valastyan S, Weinberg RA (2011). Tumor metastasis: molecular insights and evolving paradigms. Cell.

[6] Lin Y, Xu J, Lan H (2019). Tumor-associated macrophages in tumor metastasis: biological roles and clinical therapeutic applications. J Hematol Oncol.

[7] Roe JS, Hwang CI, Somerville TDD, Milazzo JP, Lee EJ, Da Silva B, Maiorino L, Tiriac H, Young CM, Miyabayashi K (2017). Enhancer reprogramming promotes pancreatic Cancer Metastasis. Cell.

[8] Dai W, Wu J, Peng X, Hou W, Huang H, Cheng Q, Liu Z, Luyten W, Schoofs L, Zhou J, Liu S (2022). CDK12 orchestrates super-enhancer-associated CCDC137 transcription to direct hepatic metastasis in colorectal cancer. Clin Transl Med.

[9] Teng S, Li YE, Yang M, Qi R, Huang Y, Wang Q, Zhang Y, Chen S, Li S, Lin K (2020). Tissue-specific transcription reprogramming promotes liver metastasis of colorectal cancer. Cell Res.

[10] Bradner JE, Hnisz D, Young RA (2017). Transcriptional addiction in Cancer. Cell.

[11] Thandapani P (2019). Super-enhancers in cancer. Pharmacol Ther.

[12] Hnisz D, Abraham BJ, Lee TI, Lau A, Saint-André V, Sigova AA, Hoke HA, Young RA (2013). Super-enhancers in the control of cell identity and disease. Cell.

[13] Wang Y, Nie H, He X, Liao Z, Zhou Y, Zhou J, Ou C (2020). The emerging role of super enhancer-derived noncoding RNAs in human cancer. Theranostics.

[14] Whyte WA, Orlando DA, Hnisz D, Abraham BJ, Lin CY, Kagey MH, Rahl PB, Lee TI, Young RA (2013). Master transcription factors and mediator establish super-enhancers at key cell identity genes. Cell.

[15] Tan Y, Li Y, Tang F (2020). Oncogenic seRNA functional activation: a novel mechanism of tumorigenesis. Mol Cancer.

[16] Tang F, Yang Z, Tan Y, Li Y (2020). Super-enhancer function and its application in cancer targeted therapy. NPJ Precis Oncol.

[17] Zhang M, Hoyle RG, Ma Z, Sun B, Cai W, Cai H, Xie N, Zhang Y, Hou J, Liu X (2021). FOSL1 promotes metastasis of head and neck squamous cell carcinoma through super-enhancer-driven transcription program. Mol Ther.

[18] Dong J, Li J, Li Y, Ma Z, Yu Y, Wang CY (2021). Transcriptional super-enhancers control cancer stemness and metastasis genes in squamous cell carcinoma. Nat Commun.

[19] Mushimiyimana I, Niskanen H, Beter M, Laakkonen JP, Kaikkonen MU, Ylä-Herttuala S, Laham-Karam N (2021). Characterization of a functional endothelial super-enhancer that regulates ADAMTS18 and angiogenesis. Nucleic Acids Res.

[20] Christensen CL, Kwiatkowski N, Abraham BJ, Carretero J, Al-Shahrour F, Zhang T, Chipumuro E, Herter-Sprie GS, Akbay EA, Altabef A (2014). Targeting transcriptional addictions in small cell lung cancer with a covalent CDK7 inhibitor. Cancer Cell.

[21] Wang Y, Zhang T, Kwiatkowski N, Abraham BJ, Lee TI, Xie S, Yuzugullu H, Von T, Li H, Lin Z (2015). CDK7-dependent transcriptional addiction in triple-negative breast cancer. Cell.

[22] Edgar Serfling MJ, Schaffner W. <enhancers and="eukaryotic=” gene="transcription.pdf=”></enhancers>. Trends Genet 1985, 1.

[23] Heintzman ND, Ren B (2009). Finding distal regulatory elements in the human genome. Curr Opin Genet Dev.

[24] Wamstad JA, Wang X, Demuren OO, Boyer LA (2014). Distal enhancers: new insights into heart development and disease. Trends Cell Biol.

[25] Hamamoto R, Takasawa K, Shinkai N, Machino H, Kouno N, Asada K, Komatsu M, Kaneko S. Analysis of super-enhancer using machine learning and its application to medical biology. Brief Bioinform 2023, 24. 10.1093/bib/bbad107 PMC10199775 36960780

[26] Hnisz D, Day DS, Young RA (2016). Insulated neighborhoods: structural and functional units of mammalian Gene Control. Cell.

[27] Hnisz D, Shrinivas K, Young RA, Chakraborty AK, Sharp PA (2017). A phase separation model for Transcriptional Control. Cell.

[28] Sabari BR, Dall’Agnese A, Boija A, Klein IA, Coffey EL, Shrinivas K, Abraham BJ, Hannett NM, Zamudio AV, Manteiga JC et al. Coactivator condensation at super-enhancers links phase separation and gene control. Science 2018, 361. 10.1126/science.aar3958 PMC6092193 29930091

[29] Fullwood MJ, Wei CL, Liu ET, Ruan Y (2009). Next-generation DNA sequencing of paired-end tags (PET) for transcriptome and genome analyses. Genome Res.

[30] Jiang Y, Harigaya Y, Zhang Z, Zhang H, Zang C, Zhang NR (2022). Nonparametric single-cell multiomic characterization of trio relationships between transcription factors, target genes, and cis-regulatory regions. Cell Syst.

[31] Wei X, Xiang Y, Peters DT, Marius C, Sun T, Shan R, Ou J, Lin X, Yue F, Li W (2022). HiCAR is a robust and sensitive method to analyze open-chromatin-associated genome organization. Mol Cell.

[32] Rossi MJ, Lai WKM, Pugh BF (2018). Simplified ChIP-exo assays. Nat Commun.

[33] Mumbach MR, Rubin AJ, Flynn RA, Dai C, Khavari PA, Greenleaf WJ, Chang HY (2016). HiChIP: efficient and sensitive analysis of protein-directed genome architecture. Nat Methods.

[34] Zheng M, Tian SZ, Capurso D, Kim M, Maurya R, Lee B, Piecuch E, Gong L, Zhu JJ, Li Z (2019). Multiplex chromatin interactions with single-molecule precision. Nature.

[35] Kaya-Okur HS, Wu SJ, Codomo CA, Pledger ES, Bryson TD, Henikoff JG, Ahmad K, Henikoff S. CUT&Tag for efficient epigenomic profiling of small samples and single cells. Nat Commun. 2019; 10:1930. 10.1038/s41467-019-09982-5 PMC6488672 31036827

[36] Xiao L, Parolia A, Qiao Y, Bawa P, Eyunni S, Mannan R, Carson SE, Chang Y, Wang X, Zhang Y (2022). Targeting SWI/SNF ATPases in enhancer-addicted prostate cancer. Nature.

[37] Zhou J, Toh SH, Tan TK, Balan K, Lim JQ, Tan TZ, Xiong S, Jia Y, Ng SB, Peng Y (2023). Super-enhancer-driven TOX2 mediates oncogenesis in natural Killer/T cell lymphoma. Mol Cancer.

[38] Cai H, Liang J, Jiang Y, Wang Z, Li H, Wang W, Wang C, Hou J (2024). KLF7 regulates super-enhancer-driven IGF2BP2 overexpression to promote the progression of head and neck squamous cell carcinoma. J Exp Clin Cancer Res.

[39] Hnisz D, Schuijers J, Lin CY, Weintraub AS, Abraham BJ, Lee TI, Bradner JE, Young RA (2015). Convergence of developmental and oncogenic signaling pathways at transcriptional super-enhancers. Mol Cell.

[40] Konermann S, Brigham MD, Trevino AE, Joung J, Abudayyeh OO, Barcena C, Hsu PD, Habib N, Gootenberg JS, Nishimasu H (2015). Genome-scale transcriptional activation by an engineered CRISPR-Cas9 complex. Nature.

[41] Thakore PI, D’Ippolito AM, Song L, Safi A, Shivakumar NK, Kabadi AM, Reddy TE, Crawford GE, Gersbach CA (2015). Highly specific epigenome editing by CRISPR-Cas9 repressors for silencing of distal regulatory elements. Nat Methods.

[42] Bahr C, von Paleske L, Uslu VV, Remeseiro S, Takayama N, Ng SW, Murison A, Langenfeld K, Petretich M, Scognamiglio R (2018). A Myc enhancer cluster regulates normal and leukaemic haematopoietic stem cell hierarchies. Nature.

[43] Li XP, Qu J, Teng XQ, Zhuang HH, Dai YH, Yang Z, Qu Q (2023). The emerging role of super-enhancers as therapeutic targets in the Digestive System tumors. Int J Biol Sci.

[44] Saint-André V, Federation AJ, Lin CY, Abraham BJ, Reddy J, Lee TI, Bradner JE, Young RA (2016). Models of human core transcriptional regulatory circuitries. Genome Res.

[45] Zhang T, Song X, Zhang Z, Mao Q, Xia W, Xu L, Jiang F, Dong G (2020). Aberrant super-enhancer landscape reveals core transcriptional regulatory circuitry in lung adenocarcinoma. Oncogenesis.

[46] Wang D, Yin Z, Wang H, Wang L, Li T, Xiao R, Xie T, Han R, Dong R, Liu H (2023). The super elongation complex drives transcriptional addiction in MYCN-amplified neuroblastoma. Sci Adv.

[47] Chipumuro E, Marco E, Christensen CL, Kwiatkowski N, Zhang T, Hatheway CM, Abraham BJ, Sharma B, Yeung C, Altabef A (2014). CDK7 inhibition suppresses super-enhancer-linked oncogenic transcription in MYCN-driven cancer. Cell.

[48] Wang QY, Peng L, Chen Y, Liao LD, Chen JX, Li M, Li YY, Qian FC, Zhang YX, Wang F (2020). Characterization of super-enhancer-associated functional lncRNAs acting as ceRNAs in ESCC. Mol Oncol.

[49] Suzuki HI, Young RA, Sharp PA (2017). Super-enhancer-mediated RNA Processing revealed by Integrative MicroRNA Network Analysis. Cell.

[50] Huang S, Li X, Zheng H, Si X, Li B, Wei G, Li C, Chen Y, Chen Y, Liao W (2019). Loss of Super-enhancer-regulated circRNA Nfix induces Cardiac Regeneration after myocardial infarction in adult mice. Circulation.

[51] Mousavi K, Zare H, Dell’orso S, Grontved L, Gutierrez-Cruz G, Derfoul A, Hager GL, Sartorelli V (2013). eRNAs promote transcription by establishing chromatin accessibility at defined genomic loci. Mol Cell.

[52] Peng L, Jiang B, Yuan X, Qiu Y, Peng J, Huang Y, Zhang C, Zhang Y, Lin Z, Li J (2019). Super-enhancer-associated long noncoding RNA HCCL5 is activated by ZEB1 and promotes the malignancy of Hepatocellular Carcinoma. Cancer Res.

[53] Zhang Y, Tan YY, Chen PP, Xu H, Xie SJ, Xu SJ, Li B, Li JH, Liu S, Yang JH (2021). Genome-wide identification of microRNA targets reveals positive regulation of the Hippo pathway by miR-122 during liver development. Cell Death Dis.

[54] Han J, Meng J, Chen S, Wang X, Yin S, Zhang Q, Liu H, Qin R, Li Z, Zhong W (2019). YY1 Complex promotes quaking expression via Super-enhancer binding during EMT of Hepatocellular Carcinoma. Cancer Res.

[55] Zhang X, Choi PS, Francis JM, Imielinski M, Watanabe H, Cherniack AD, Meyerson M (2016). Identification of focally amplified lineage-specific super-enhancers in human epithelial cancers. Nat Genet.

[56] Oldridge DA, Wood AC, Weichert-Leahey N, Crimmins I, Sussman R, Winter C, McDaniel LD, Diamond M, Hart LS, Zhu S (2015). Genetic predisposition to neuroblastoma mediated by a LMO1 super-enhancer polymorphism. Nature.

[57] Mansour MR, Abraham BJ, Anders L, Berezovskaya A, Gutierrez A, Durbin AD, Etchin J, Lawton L, Sallan SE, Silverman LB (2014). Oncogene regulation. An oncogenic super-enhancer formed through somatic mutation of a noncoding intergenic element. Science.

[58] Jia Q, Chen S, Tan Y, Li Y, Tang F (2020). Oncogenic super-enhancer formation in tumorigenesis and its molecular mechanisms. Exp Mol Med.

[59] Merkenschlager M, Nora EP (2016). CTCF and Cohesin in genome folding and transcriptional gene regulation. Annu Rev Genomics Hum Genet.

[60] Weischenfeldt J, Dubash T, Drainas AP, Mardin BR, Chen Y, Stütz AM, Waszak SM, Bosco G, Halvorsen AR, Raeder B (2017). Pan-cancer analysis of somatic copy-number alterations implicates IRS4 and IGF2 in enhancer hijacking. Nat Genet.

[61] Flavahan WA, Drier Y, Liau BB, Gillespie SM, Venteicher AS, Stemmer-Rachamimov AO, Suvà ML, Bernstein BE (2016). Insulator dysfunction and oncogene activation in IDH mutant gliomas. Nature.

[62] Nakagawa M, Shaffer AL, Ceribelli M, Zhang M, Wright G, Huang DW, Xiao W, Powell J, Petrus MN, Yang Y (2018). Targeting the HTLV-I-Regulated BATF3/IRF4 Transcriptional Network in Adult T Cell Leukemia/Lymphoma. Cancer Cell.

[63] Daenthanasanmak A, Bamford RN, Yoshioka M, Yang SM, Homan P, Karim B, Bryant BR, Petrus MN, Thomas CJ, Green PL (2022). Triple combination of BET plus PI3K and NF-κB inhibitors exhibit synergistic activity in adult T-cell leukemia/lymphoma. Blood Adv.

[64] Zhou H, Schmidt SC, Jiang S, Willox B, Bernhardt K, Liang J, Johannsen EC, Kharchenko P, Gewurz BE, Kieff E, Zhao B (2015). Epstein-Barr virus oncoprotein super-enhancers control B cell growth. Cell Host Microbe.

[65] Gunnell A, Webb HM, Wood CD, McClellan MJ, Wichaidit B, Kempkes B, Jenner RG, Osborne C, Farrell PJ, West MJ (2016). RUNX super-enhancer control through the notch pathway by Epstein-Barr virus transcription factors regulates B cell growth. Nucleic Acids Res.

[66] Yan B, Wang C, Chakravorty S, Zhang Z, Kadadi SD, Zhuang Y, Sirit I, Hu Y, Jung M, Sahoo SS (2023). A comprehensive single cell data analysis of lymphoblastoid cells reveals the role of super-enhancers in maintaining EBV latency. J Med Virol.

[67] Dębek S, Juszczyński P (2022). Super enhancers as master gene regulators in the pathogenesis of hematologic malignancies. Biochim Biophys Acta Rev Cancer.

[68] Nakamura Y, Hattori N, Iida N, Yamashita S, Mori A, Kimura K, Yoshino T, Ushijima T (2017). Targeting of super-enhancers and mutant BRAF can suppress growth of BRAF-mutant colon cancer cells via repression of MAPK signaling pathway. Cancer Lett.

[69] Herranz D, Ambesi-Impiombato A, Palomero T, Schnell SA, Belver L, Wendorff AA, Xu L, Castillo-Martin M, Llobet-Navás D, Cordon-Cardo C (2014). A NOTCH1-driven MYC enhancer promotes T cell development, transformation and acute lymphoblastic leukemia. Nat Med.

[70] Zheng ZZ, Xia L, Hu GS, Liu JY, Hu YH, Chen YJ, Peng JY, Zhang WJ, Liu W (2022). Super-enhancer-controlled positive feedback loop BRD4/ERα-RET-ERα promotes ERα-positive breast cancer. Nucleic Acids Res.

[71] Hargreaves DC, Crabtree GR (2011). ATP-dependent chromatin remodeling: genetics, genomics and mechanisms. Cell Res.

[72] Shi J, Whyte WA, Zepeda-Mendoza CJ, Milazzo JP, Shen C, Roe JS, Minder JL, Mercan F, Wang E, Eckersley-Maslin MA (2013). Role of SWI/SNF in acute leukemia maintenance and enhancer-mediated myc regulation. Genes Dev.

[73] Li X, Zhu R, Jiang H, Yin Q, Gu J, Chen J, Ji X, Wu X, Fu H, Wang H (2022). Autophagy enhanced by curcumin ameliorates inflammation in atherogenesis via the TFEB-P300-BRD4 axis. Acta Pharm Sin B.

[74] Wagh K, Garcia DA, Upadhyaya A (2021). Phase separation in transcription factor dynamics and chromatin organization. Curr Opin Struct Biol.

[75] Kainth AS, Chowdhary S, Pincus D, Gross DS (2021). Primordial super-enhancers: heat shock-induced chromatin organization in yeast. Trends Cell Biol.

[76] Boija A, Klein IA, Sabari BR, Dall’Agnese A, Coffey EL, Zamudio AV, Li CH, Shrinivas K, Manteiga JC, Hannett NM (2018). Transcription factors activate genes through the phase-separation capacity of their activation domains. Cell.

[77] Ahn JH, Davis ES, Daugird TA, Zhao S, Quiroga IY, Uryu H, Li J, Storey AJ, Tsai YH, Keeley DP (2021). Phase separation drives aberrant chromatin looping and cancer development. Nature.

[78] Patel SA, Rodrigues P, Wesolowski L, Vanharanta S (2021). Genomic control of metastasis. Br J Cancer.

[79] McDonald OG, Li X, Saunders T, Tryggvadottir R, Mentch SJ, Warmoes MO, Word AE, Carrer A, Salz TH, Natsume S (2017). Epigenomic reprogramming during pancreatic cancer progression links anabolic glucose metabolism to distant metastasis. Nat Genet.

[80] Kim EJ, Liu P, Zhang S, Donahue K, Wang Y, Schehr JL, Wolfe SK, Dickerson A, Lu L, Rui L (2021). BAF155 methylation drives metastasis by hijacking super-enhancers and subverting anti-tumor immunity. Nucleic Acids Res.

[81] Yu D, Yang X, Lin J, Cao Z, Lu C, Yang Z, Zheng M, Pan R, Cai W (2021). Super-enhancer Induced IL-20RA promotes Proliferation/Metastasis and Immune Evasion in Colorectal Cancer. Front Oncol.

[82] Rojas-Puentes L, Cardona AF, Carranza H, Vargas C, Jaramillo LF, Zea D, Cetina L, Wills B, Ruiz-Garcia E, Arrieta O (2016). Epithelial-mesenchymal transition, proliferation, and angiogenesis in locally advanced cervical cancer treated with chemoradiotherapy. Cancer Med.

[83] Zheng X, Carstens JL, Kim J, Scheible M, Kaye J, Sugimoto H, Wu CC, LeBleu VS, Kalluri R (2015). Epithelial-to-mesenchymal transition is dispensable for metastasis but induces chemoresistance in pancreatic cancer. Nature.

[84] Suhail Y, Cain MP, Vanaja K, Kurywchak PA, Levchenko A, Kalluri R (2019). Kshitiz: systems Biology of Cancer Metastasis. Cell Syst.

[85] Ri K, Kim C, Pak C, Ri P, Om H (2020). The KLF6 super enhancer modulates cell proliferation via MiR-1301 in human hepatoma cells. Microrna.

[86] Cui S, Wu Q, Liu M, Su M, Liu S, Shao L, Han X, He H (2021). EphA2 super-enhancer promotes tumor progression by recruiting FOSL2 and TCF7L2 to activate the target gene EphA2. Cell Death Dis.

[87] Jiang Y, Jiang YY, Xie JJ, Mayakonda A, Hazawa M, Chen L, Xiao JF, Li CQ, Huang ML, Ding LW (2018). Co-activation of super-enhancer-driven CCAT1 by TP63 and SOX2 promotes squamous cancer progression. Nat Commun.

[88] Xiang JF, Yin QF, Chen T, Zhang Y, Zhang XO, Wu Z, Zhang S, Wang HB, Ge J, Lu X (2014). Human colorectal cancer-specific CCAT1-L lncRNA regulates long-range chromatin interactions at the MYC locus. Cell Res.

[89] Zhang T, Xia W, Song X, Mao Q, Huang X, Chen B, Liang Y, Wang H, Chen Y, Yu X (2022). Super-enhancer hijacking LINC01977 promotes malignancy of early-stage lung adenocarcinoma addicted to the canonical TGF-β/SMAD3 pathway. J Hematol Oncol.

[90] Dai W, Guo C, Wang Y, Li Y, Xie R, Wu J, Yao B, Xie D, He L, Li Y (2023). Identification of hub genes and pathways in lung metastatic colorectal cancer. BMC Cancer.

[91] Chen Y, Song Y, Du W, Gong L, Chang H, Zou Z (2019). Tumor-associated macrophages: an accomplice in solid tumor progression. J Biomed Sci.

[92] Li QL, Lin X, Yu YL, Chen L, Hu QX, Chen M, Cao N, Zhao C, Wang CY, Huang CW (2021). Genome-wide profiling in colorectal cancer identifies PHF19 and TBC1D16 as oncogenic super enhancers. Nat Commun.

[93] Ying Y, Wang Y, Huang X, Sun Y, Zhang J, Li M, Zeng J, Wang M, Xiao W, Zhong L (2020). Oncogenic HOXB8 is driven by MYC-regulated super-enhancer and potentiates colorectal cancer invasiveness via BACH1. Oncogene.

[94] Xie JJ, Jiang YY, Jiang Y, Li CQ, Lim MC, An O, Mayakonda A, Ding LW, Long L, Sun C (2018). Super-enhancer-driven Long non-coding RNA LINC01503, regulated by TP63, is over-expressed and oncogenic in squamous cell carcinoma. Gastroenterology.

[95] Xu S, Wan L, Yin H, Xu H, Zheng W, Shen M, Zhang Z, Pang D (2017). Long noncoding RNA Linc00152 functions as a Tumor Propellant in Pan-cancer. Cell Physiol Biochem.

[96] Deng R, Huang JH, Wang Y, Zhou LH, Wang ZF, Hu BX, Chen YH, Yang D, Mai J, Li ZL (2020). Disruption of super-enhancer-driven tumor suppressor gene RCAN1.4 expression promotes the malignancy of breast carcinoma. Mol Cancer.

[97] Doustmihan A, Fathi M, Mazloomi M, Salemi A, Hamblin MR, Jahanban-Esfahlan R. Molecular targets, therapeutic agents and multitasking nanoparticles to deal with cancer stem cells: a narrative review. J Control Release 2023. 10.1016/j.jconrel.2023.09.029 37739017

[98] Zhou J, Wang S, Nie D, Lai P, Li Y, Li Y, Jin Y, Pan J. Super-enhancer landscape reveals leukemia stem cell reliance on X-box binding protein 1 as a therapeutic vulnerability. 2021, 13:eabh3462. 10.1126/scitranslmed.abh3462 34550724

[99] Caslini C, Hong S, Ban YJ, Chen XS, Ince TA (2019). HDAC7 regulates histone 3 lysine 27 acetylation and transcriptional activity at super-enhancer-associated genes in breast cancer stem cells. Oncogene.

[100] Blackman DJ, Masi CM (2006). Racial and ethnic disparities in breast cancer mortality: are we doing enough to address the root causes?. J Clin Oncol.

[101] Xing F, Zhao D, Wu SY, Tyagi A, Wu K, Sharma S, Liu Y, Deshpande R, Wang Y, Cleary J (2021). Epigenetic and posttranscriptional modulation of SOS1 can promote breast Cancer metastasis through obesity-activated c-Met Signaling in African-American Women. Cancer Res.

[102] Chen T, Tang X, Wang Z, Feng F, Xu C, Zhao Q, Wu Y, Sun H, Chen Y (2023). Inhibition of Son of Sevenless Homologue 1 (SOS1): promising therapeutic treatment for KRAS-mutant cancers. Eur J Med Chem.

[103] Ang HL, Mohan CD, Shanmugam MK, Leong HC, Makvandi P, Rangappa KS, Bishayee A, Kumar AP, Sethi G (2023). Mechanism of epithelial-mesenchymal transition in cancer and its regulation by natural compounds. Med Res Rev.

[104] Su T, Zhang N, Wang T, Zeng J, Li W, Han L, Yang M (2023). Super enhancer-regulated LncRNA LINC01089 induces alternative splicing of DIAPH3 to Drive Hepatocellular Carcinoma Metastasis. Cancer Res.

[105] Yang Z, Zheng Y, Wu H, Xie H, Zhao J, Chen Z, Li L, Yue X, Zhao B, Bian E (2023). Integrative analysis of a novel super-enhancer-associated lncRNA prognostic signature and identifying LINC00945 in aggravating glioma progression. Hum Genomics.

[106] Zhu X, Zhang T, Zhang Y, Chen H, Shen J, Jin X, Wei J, Zhang E, Xiao M, Fan Y (2020). A super-enhancer controls TGF- β signaling in pancreatic cancer through downregulation of TGFBR2. Cell Signal.

[107] Yang WL, Wang J, Chan CH, Lee SW, Campos AD, Lamothe B, Hur L, Grabiner BC, Lin X, Darnay BG, Lin HK (2009). The E3 ligase TRAF6 regulates akt ubiquitination and activation. Science.

[108] Zhang C, Wei S, Sun WP, Teng K, Dai MM, Wang FW, Chen JW, Ling H, Ma XD, Feng ZH (2020). Super-enhancer-driven AJUBA is activated by TCF4 and involved in epithelial-mesenchymal transition in the progression of Hepatocellular Carcinoma. Theranostics.

[109] Liu L, Dai Y, Chen J, Zeng T, Li Y, Chen L, Zhu YH, Li J, Li Y, Ma S (2014). Maelstrom promotes hepatocellular carcinoma metastasis by inducing epithelial-mesenchymal transition by way of Akt/GSK-3β/Snail signaling. Hepatology.

[110] Wang W, Yun B, Hoyle RG, Ma Z, Zaman SU, Xiong G, Yi C, Xie N, Zhang M, Liu X (2024). CYTOR facilitates formation of FOSL1 phase separation and Super enhancers to drive metastasis of Tumor budding cells in Head and Neck squamous cell carcinoma. Adv Sci (Weinh).

[111] Kothapalli D, Flowers J, Xu T, Puré E, Assoian RK (2008). Differential activation of ERK and Rac mediates the proliferative and anti-proliferative effects of hyaluronan and CD44. J Biol Chem.

[112] Ren B, Cui M, Yang G, Wang H, Feng M, You L, Zhao Y (2018). Tumor microenvironment participates in metastasis of pancreatic cancer. Mol Cancer.

[113] Quail DF, Joyce JA (2013). Microenvironmental regulation of tumor progression and metastasis. Nat Med.

[114] Wu F, Yang J, Liu J, Wang Y, Mu J, Zeng Q, Deng S, Zhou H (2021). Signaling pathways in cancer-associated fibroblasts and targeted therapy for cancer. Signal Transduct Target Ther.

[115] Deepak KGK, Vempati R, Nagaraju GP, Dasari VR, Rao SN, Malla DN (2020). Tumor microenvironment: challenges and opportunities in targeting metastasis of triple negative breast cancer. Pharmacol Res.

[116] Pitt JM, Marabelle A, Eggermont A, Soria JC, Kroemer G, Zitvogel L (2016). Targeting the tumor microenvironment: removing obstruction to anticancer immune responses and immunotherapy. Ann Oncol.

[117] Nishida J, Momoi Y, Miyakuni K, Tamura Y, Takahashi K, Koinuma D, Miyazono K, Ehata S (2020). Epigenetic remodelling shapes inflammatory renal cancer and neutrophil-dependent metastasis. Nat Cell Biol.

[118] Zhou RW, Xu J, Martin TC, Zachem AL, He J, Ozturk S, Demircioglu D, Bansal A, Trotta AP, Giotti B (2022). A local tumor microenvironment acquired super-enhancer induces an oncogenic driver in colorectal carcinoma. Nat Commun.

[119] Wellenstein MD, de Visser KE (2018). Cancer-Cell-intrinsic mechanisms shaping the Tumor Immune Landscape. Immunity.

[120] Vinay DS, Ryan EP, Pawelec G, Talib WH, Stagg J, Elkord E, Lichtor T, Decker WK, Whelan RL, Kumara H (2015). Immune evasion in cancer: mechanistic basis and therapeutic strategies. Semin Cancer Biol.

[121] Goodman A, Patel SP, Kurzrock R (2017). PD-1-PD-L1 immune-checkpoint blockade in B-cell lymphomas. Nat Rev Clin Oncol.

[122] Xu Y, Wu Y, Zhang S, Ma P, Jin X, Wang Z, Yao M, Zhang E, Tao B, Qin Y (2019). A tumor-specific Super-enhancer drives Immune Evasion by Guiding Synchronous expression of PD-L1 and PD-L2. Cell Rep.

[123] Feng Q, Liu Z, Yu X, Huang T, Chen J, Wang J, Wilhelm J, Li S, Song J, Li W (2022). Lactate increases stemness of CD8 + T cells to augment anti-tumor immunity. Nat Commun.

[124] Duan Q, Mao X, Xiao Y, Liu Z, Wang Y, Zhou H, Zhou Z, Cai J, Xia K, Zhu Q (2016). Super enhancers at the miR-146a and miR-155 genes contribute to self-regulation of inflammation. Biochim Biophys Acta.

[125] Yin C, Han Q, Xu D, Zheng B, Zhao X, Zhang J (2019). SALL4-mediated upregulation of exosomal miR-146a-5p drives T-cell exhaustion by M2 tumor-associated macrophages in HCC. Oncoimmunology.

[126] Li X, Wang S, Mu W, Barry J, Han A, Carpenter RL, Jiang BH, Peiper SC, Mahoney MG, Aplin AE (2022). Reactive oxygen species reprogram macrophages to suppress antitumor immune response through the exosomal miR-155-5p/PD-L1 pathway. J Exp Clin Cancer Res.

[127] Akbarian M, Bertassoni LE, Tayebi L (2022). Biological aspects in controlling angiogenesis: current progress. Cell Mol Life Sci.

[128] Raza A, Franklin MJ, Dudek AZ (2010). Pericytes and vessel maturation during tumor angiogenesis and metastasis. Am J Hematol.

[129] Paoluzzi L, Maki RG (2019). Diagnosis, prognosis, and treatment of alveolar soft-part sarcoma: a review. JAMA Oncol.

[130] Jaber OI, Kirby PA (2015). Alveolar soft part sarcoma. Arch Pathol Lab Med.

[131] Tanaka M, Chuaychob S, Homme M, Yamazaki Y, Lyu R, Yamashita K, Ae K, Matsumoto S, Kumegawa K, Maruyama R et al. ASPSCR1::TFE3 orchestrates the angiogenic program of alveolar soft part sarcoma. *Nat Commun* 2023, 14:1957. 10.1038/s41467-023-37049-z PMC10082046 37029109

[132] Nguyen DT, Yang W, Renganathan A, Weimholt C, Angappulige DH, Nguyen T, Sprung RW, Andriole GL, Kim EH, Mahajan NP, Mahajan K (2022). Acetylated HOXB13 regulated super enhancer genes define therapeutic vulnerabilities of castration-resistant prostate Cancer. Clin Cancer Res.

[133] Zhang Q, Yu S, Lam MMT, Poon TCW, Sun L, Jiao Y, Wong AST, Lee LTO (2019). Angiotensin II promotes ovarian cancer spheroid formation and metastasis by upregulation of lipid desaturation and suppression of endoplasmic reticulum stress. J Exp Clin Cancer Res.

[134] Takiguchi T, Takahashi-Yanaga F, Ishikane S, Tetsuo F, Hosoda H, Arioka M, Kitazono T, Sasaguri T (2021). Angiotensin II promotes primary tumor growth and metastasis formation of murine TNBC 4T1 cells through the fibroblasts around cancer cells. Eur J Pharmacol.

[135] Zhang HF, Gao X, Wang X, Chen X, Huang Y, Wang L, Xu ZW (2021). The mechanisms of renin-angiotensin system in hepatocellular carcinoma: from the perspective of liver fibrosis, HCC cell proliferation, metastasis and angiogenesis, and corresponding protection measures. Biomed Pharmacother.

[136] Das S, Senapati P, Chen Z, Reddy MA, Ganguly R, Lanting L, Mandi V, Bansal A, Leung A, Zhang S (2017). Regulation of angiotensin II actions by enhancers and super-enhancers in vascular smooth muscle cells. Nat Commun.

[137] Sleeboom JJF, van Tienderen GS, Schenke-Layland K, van der Laan LJW, Khalil AA, Verstegen MMA (2024). The extracellular matrix as hallmark of cancer and metastasis: from biomechanics to therapeutic targets. Sci Transl Med.

[138] Winkler J, Abisoye-Ogunniyan A, Metcalf KJ, Werb Z (2020). Concepts of extracellular matrix remodelling in tumour progression and metastasis. Nat Commun.

[139] Zhou C, York SR, Chen JY, Pondick JV, Motola DL, Chung RT, Mullen AC (2016). Long noncoding RNAs expressed in human hepatic stellate cells form networks with extracellular matrix proteins. Genome Med.

[140] Dai W, Liu S, Wang S, Zhao L, Yang X, Zhou J, Wang Y, Zhang J, Zhang P, Ding K (2021). Activation of transmembrane receptor tyrosine kinase DDR1-STAT3 cascade by extracellular matrix remodeling promotes liver metastatic colonization in uveal melanoma. Signal Transduct Target Ther.

[141] Zhao S, Mi Y, Zheng B, Wei P, Gu Y, Zhang Z, Xu Y, Cai S, Li X, Li D (2022). Highly-metastatic colorectal cancer cell released miR-181a-5p-rich extracellular vesicles promote liver metastasis by activating hepatic stellate cells and remodelling the tumour microenvironment. J Extracell Vesicles.

[142] Wang Y, Chen K, Liu G, Du C, Cheng Z, Wei D, Li F, Li C, Yang Y, Zhao Y, Nie G. Disruption of super-enhancers in activated pancreatic stellate cells facilitates Chemotherapy and Immunotherapy in Pancreatic Cancer. Adv Sci (Weinh) 2024:e2308637. 10.1002/advs.202308637 PMC11040371 38417121

[143] Lee KW, Yeo SY, Gong JR, Koo OJ, Sohn I, Lee WY, Kim HC, Yun SH, Cho YB, Choi MA (2022). PRRX1 is a master transcription factor of stromal fibroblasts for myofibroblastic lineage progression. Nat Commun.

[144] Wang W, Tang YA, Xiao Q, Lee WC, Cheng B, Niu Z, Oguz G, Feng M, Lee PL, Li B (2021). Stromal induction of BRD4 phosphorylation results in chromatin remodeling and BET inhibitor resistance in Colorectal Cancer. Nat Commun.

[145] Guo J, Zheng Q, Peng Y (2023). BET proteins: Biological functions and therapeutic interventions. Pharmacol Ther.

[146] Bacabac M, Xu W (2023). Oncogenic super-enhancers in cancer: mechanisms and therapeutic targets. Cancer Metastasis Rev.

[147] Schwalm MP, Knapp S (2022). BET bromodomain inhibitors. Curr Opin Chem Biol.

[148] Wahi A, Manchanda N, Jain P, Jadhav HR (2023). Targeting the epigenetic reader BET as a therapeutic strategy for cancer. Bioorg Chem.

[149] Chen D, Zhao Z, Huang Z, Chen DC, Zhu XX, Wang YZ, Yan YW, Tang S, Madhavan S, Ni W (2018). Super enhancer inhibitors suppress MYC driven transcriptional amplification and tumor progression in osteosarcoma. Bone Res.

[150] Liu SX, Wang C, Lin RB, Ding WY, Roy G, Wang HB, Yang T, Liu Q, Luo YL, Jin SL (2023). Super-enhancer driven SOX2 promotes tumor formation by chromatin re-organization in nasopharyngeal carcinoma. EBioMedicine.

[151] Ott CJ, Federation AJ, Schwartz LS, Kasar S, Klitgaard JL, Lenci R, Li Q, Lawlor M, Fernandes SM, Souza A (2018). Enhancer Architecture and Essential Core Regulatory Circuitry of Chronic Lymphocytic Leukemia. Cancer Cell.

[152] Fong CY, Gilan O, Lam EY, Rubin AF, Ftouni S, Tyler D, Stanley K, Sinha D, Yeh P, Morison J (2015). BET inhibitor resistance emerges from leukaemia stem cells. Nature.

[153] Nieto-Jiménez C, Morafraile EC, Alonso-Moreno C, Ocaña A (2022). Clinical considerations for the design of PROTACs in cancer. Mol Cancer.

[154] Ma Z, Bolinger AA, Zhou J, Tian B (2023). Bromodomain-containing protein 4 (BRD4): a key player in inflammatory bowel disease and potential to inspire epigenetic therapeutics. Expert Opin Ther Targets.

[155] Jia SQ, Zhuo R, Zhang ZM, Yang Y, Tao YF, Wang JW, Li XL, Xie Y, Li G, Wu D et al. The BRD4 inhibitor dBET57 exerts anticancer effects by targeting superenhancer-related genes in neuroblastoma. J Immunol Res. 2022;2022:7945884. 10.1155/2022/7945884 PMC9691391 36438198

[156] Winter GE, Mayer A, Buckley DL, Erb MA, Roderick JE, Vittori S, Reyes JM, di Iulio J, Souza A, Ott CJ (2017). BET Bromodomain Proteins Function as Master transcription elongation factors Independent of CDK9 Recruitment. Mol Cell.

[157] Raina K, Lu J, Qian Y, Altieri M, Gordon D, Rossi AM, Wang J, Chen X, Dong H, Siu K (2016). PROTAC-induced BET protein degradation as a therapy for castration-resistant prostate cancer. Proc Natl Acad Sci U S A.

[158] Zhou B, Hu J, Xu F, Chen Z, Bai L, Fernandez-Salas E, Lin M, Liu L, Yang CY, Zhao Y (2018). Discovery of a small-molecule degrader of Bromodomain and Extra-terminal (BET) proteins with Picomolar Cellular potencies and capable of achieving Tumor Regression. J Med Chem.

[159] Lu J, Qian Y, Altieri M, Dong H, Wang J, Raina K, Hines J, Winkler JD, Crew AP, Coleman K, Crews CM (2015). Hijacking the E3 Ubiquitin Ligase Cereblon to efficiently target BRD4. Chem Biol.

[160] Piya S, Yang Y, Bhattacharya S, Sharma P, Ma H, Mu H, He H, Ruvolo V, Baran N, Davis RE (2022). Targeting the NOTCH1-MYC-CD44 axis in leukemia-initiating cells in T-ALL. Leukemia.

[161] Piya S, Bhattacharya S, Mu H, Lorenzi PL, McQueen T, Davis ER, Ruvolo V, Baran N, Qian Y, Crews C (2016). BRD4 Proteolysis Targeting Chimera (PROTAC) ARV-825, causes sustained degradation of BRD4 and modulation of chemokine receptors, cell adhesion and metabolic targets in Leukemia resulting in Profound Anti-leukemic effects. Blood.

[162] Nowak RP, DeAngelo SL, Buckley D, He Z, Donovan KA, An J, Safaee N, Jedrychowski MP, Ponthier CM, Ishoey M (2018). Plasticity in binding confers selectivity in ligand-induced protein degradation. Nat Chem Biol.

[163] Winter GE, Buckley DL, Paulk J, Roberts JM, Souza A, Dhe-Paganon S, Bradner JE (2015). DRUG DEVELOPMENT. Phthalimide conjugation as a strategy for in vivo target protein degradation. Science.

[164] Mughal MJ, Bhadresha K, Kwok HF (2023). CDK inhibitors from past to present: a new wave of cancer therapy. Semin Cancer Biol.

[165] Ghafouri-Fard S, Khoshbakht T, Hussen BM, Dong P, Gassler N, Taheri M, Baniahmad A, Dilmaghani NA (2022). A review on the role of cyclin dependent kinases in cancers. Cancer Cell Int.

[166] Xie Z, Hou S, Yang X, Duan Y, Han J, Wang Q, Liao C (2022). Lessons learned from past cyclin-dependent kinase drug Discovery efforts. J Med Chem.

[167] Sava GP, Fan H, Coombes RC, Buluwela L, Ali S (2020). CDK7 inhibitors as anticancer drugs. Cancer Metastasis Rev.

[168] Kovalová M, Baraka JP, Mik V, Jorda R, Luo L, Shao H, Kryštof V (2023). A patent review of cyclin-dependent kinase 7 (CDK7) inhibitors (2018–2022). Expert Opin Ther Pat.

[169] Wu D, Zhang Z, Chen X, Yan Y, Liu X (2021). Angel or Devil ? - CDK8 as the new drug target. Eur J Med Chem.

[170] Dannappel MV, Sooraj D, Loh JJ, Firestein R (2018). Molecular and in vivo functions of the CDK8 and CDK19 kinase modules. Front Cell Dev Biol.

[171] Chou J, Quigley DA, Robinson TM, Feng FY, Ashworth A (2020). Transcription-Associated cyclin-dependent kinases as targets and biomarkers for Cancer Therapy. Cancer Discov.

[172] Wei Y, Li C, Bian H, Qian W, Jin K, Xu T, Guo X, Lu X, Su F (2021). Targeting CDK7 suppresses super enhancer-linked inflammatory genes and alleviates CAR T cell-induced cytokine release syndrome. Mol Cancer.

[173] Zhang J, Liu W, Zou C, Zhao Z, Lai Y, Shi Z, Xie X, Huang G, Wang Y, Zhang X (2020). Targeting super-enhancer-associated oncogenes in Osteosarcoma with THZ2, a covalent CDK7 inhibitor. Clin Cancer Res.

[174] Huang L, Yang H, Chen K, Yuan J, Li J, Dai G, Gu M, Shi Y (2023). The suppressive efficacy of THZ1 depends on KRAS mutation subtype and is associated with super-enhancer activity and the PI3K/AKT/mTOR signalling in pancreatic ductal adenocarcinoma: a hypothesis-generating study. Clin Transl Med.

[175] Hu C, Shen L, Zou F, Wu Y, Wang B, Wang A, Wu C, Wang L, Liu J, Wang W, Liu Q (2023). Predicting and overcoming resistance to CDK9 inhibitors for cancer therapy. Acta Pharm Sin B.

[176] Thieme E, Bruss N, Sun D, Dominguez EC, Coleman D, Liu T, Roleder C, Martinez M, Garcia-Mansfield K, Ball B (2023). CDK9 inhibition induces epigenetic reprogramming revealing strategies to circumvent resistance in lymphoma. Mol Cancer.

[177] Pelish HE, Liau BB, Nitulescu II, Tangpeerachaikul A, Poss ZC, Da Silva DH, Caruso BT, Arefolov A, Fadeyi O, Christie AL (2015). Mediator kinase inhibition further activates super-enhancer-associated genes in AML. Nature.

[178] Zhang T, Kwiatkowski N, Olson CM, Dixon-Clarke SE, Abraham BJ, Greifenberg AK, Ficarro SB, Elkins JM, Liang Y, Hannett NM (2016). Covalent targeting of remote cysteine residues to develop CDK12 and CDK13 inhibitors. Nat Chem Biol.

[179] Lee JM, Hammarén HM, Savitski MM, Baek SH (2023). Control of protein stability by post-translational modifications. Nat Commun.

[180] Li GH, Qu Q, Qi TT, Teng XQ, Zhu HH, Wang JJ, Lu Q, Qu J (2021). Super-enhancers: a new frontier for epigenetic modifiers in cancer chemoresistance. J Exp Clin Cancer Res.

[181] Dorna D, Grabowska A, Paluszczak J. Natural products modulating epigenetic mechanisms by affecting histone methylation/demethylation: targeting cancer cells. Br J Pharmacol 2023. 10.1111/bph.16237 37700551

[182] Li M, Liu M, Han W, Wang Z, Han D, Patalano S, Macoska JA, Balk SP, He HH, Corey E (2023). LSD1 inhibition disrupts Super-enhancer-driven Oncogenic Transcriptional Programs in Castration-resistant prostate Cancer. Cancer Res.

[183] Fagan RJ, Dingwall AK (2019). COMPASS ascending: emerging clues regarding the roles of MLL3/KMT2C and MLL2/KMT2D proteins in cancer. Cancer Lett.

[184] Alam H, Tang M, Maitituoheti M, Dhar SS, Kumar M, Han CY, Ambati CR, Amin SB, Gu B, Chen TY (2020). KMT2D Deficiency impairs super-enhancers to Confer a glycolytic vulnerability in Lung Cancer. Cancer Cell.

[185] Andricovich J, Perkail S, Kai Y, Casasanta N, Peng W, Tzatsos A (2018). Loss of KDM6A activates super-enhancers to induce gender-specific squamous-like pancreatic Cancer and confers sensitivity to BET inhibitors. Cancer Cell.

[186] Zhang J, Ying Y, Li M, Wang M, Huang X, Jia M, Zeng J, Ma C, Zhang Y, Li C (2020). Targeted inhibition of KDM6 histone demethylases eradicates tumor-initiating cells via enhancer reprogramming in colorectal cancer. Theranostics.

[187] Wiese M, Hamdan FH, Kubiak K, Diederichs C, Gielen GH, Nussbaumer G, Carcaboso AM, Hulleman E, Johnsen SA, Kramm CM (2020). Combined treatment with CBP and BET inhibitors reverses inadvertent activation of detrimental super enhancer programs in DIPG cells. Cell Death Dis.

[188] Nguyen TTT, Zhang Y, Shang E, Shu C, Torrini C, Zhao J, Bianchetti E, Mela A, Humala N, Mahajan A (2020). HDAC inhibitors elicit metabolic reprogramming by targeting super-enhancers in glioblastoma models. J Clin Invest.

[189] Sengupta S, George RE (2017). Super-enhancer-driven Transcriptional dependencies in Cancer. Trends Cancer.

[190] Okabe A, Kaneda A (2021). Transcriptional dysregulation by aberrant enhancer activation and rewiring in cancer. Cancer Sci.

[191] Gupta R, Mehta A, Wajapeyee N (2022). Transcriptional determinants of cancer immunotherapy response and resistance. Trends Cancer.

[192] Liu S, He L, Wu J, Wu X, Xie L, Dai W, Chen L, Xie F, Liu Z (2021). DHX9 contributes to the malignant phenotypes of colorectal cancer via activating NF-κB signaling pathway. Cell Mol Life Sci.

[193] Adam RC, Yang H, Rockowitz S, Larsen SB, Nikolova M, Oristian DS, Polak L, Kadaja M, Asare A, Zheng D, Fuchs E (2015). Pioneer factors govern super-enhancer dynamics in stem cell plasticity and lineage choice. Nature.

[194] Yi M, Tan Y, Wang L, Cai J, Li X, Zeng Z, Xiong W, Li G, Li X, Tan P, Xiang B (2020). TP63 links chromatin remodeling and enhancer reprogramming to epidermal differentiation and squamous cell carcinoma development. Cell Mol Life Sci.

[195] Jiang G, Wang X, Sheng D, Zhou L, Liu Y, Xu C, Liu S, Zhang J (2019). Cooperativity of co-factor NR2F2 with Pioneer factors GATA3, FOXA1 in promoting ERα function. Theranostics.

[196] Iwafuchi-Doi M, Zaret KS (2014). Pioneer transcription factors in cell reprogramming. Genes Dev.

[197] Balsalobre A, Drouin J (2022). Pioneer factors as master regulators of the epigenome and cell fate. Nat Rev Mol Cell Biol.

[198] Teng M, Zhou S, Cai C, Lupien M, He HH (2021). Pioneer of prostate cancer: past, present and the future of FOXA1. Protein Cell.

[199] Jozwik KM, Carroll JS (2012). Pioneer factors in hormone-dependent cancers. Nat Rev Cancer.

[200] Darnell JE (2002). Transcription factors as targets for cancer therapy. Nat Rev Cancer.

[201] Li Y, Song J, Zhou P, Zhou J, Xie S (2022). Targeting undruggable transcription factors with PROTACs: advances and perspectives. J Med Chem.

[202] Starodub A, Gollerkeri A, savi CD, Dey J, Agarwal S, Donohue S, Perea R, Klaus C, Gollob J. Phase 1 study of KT-333, a targeted protein degrader, in patients with relapsed or refractory lymphomas, large granular lymphocytic leukemia, and solid tumors. 2022, 40:TPS3171–3171.

[203] Proof-of (2020). Concept with PROTACs in prostate Cancer. Cancer Discov.

[204] Qi SM, Dong J, Xu ZY, Cheng XD, Zhang WD, Qin JJ (2021). PROTAC: an effective targeted protein degradation strategy for Cancer Therapy. Front Pharmacol.

[205] Zhan T, Rindtorff N, Betge J, Ebert MP, Boutros M (2019). CRISPR/Cas9 for cancer research and therapy. Semin Cancer Biol.

[206] Chen M, Mao A, Xu M, Weng Q, Mao J, Ji J (2019). CRISPR-Cas9 for cancer therapy: opportunities and challenges. Cancer Lett.

[207] Wang SW, Gao C, Zheng YM, Yi L, Lu JC, Huang XY, Cai JB, Zhang PF, Cui YH, Ke AW (2022). Current applications and future perspective of CRISPR/Cas9 gene editing in cancer. Mol Cancer.

[208] Jiang YY, Jiang Y, Li CQ, Zhang Y, Dakle P, Kaur H, Deng JW, Lin RY, Han L, Xie JJ (2020). TP63, SOX2, and KLF5 establish a Core Regulatory Circuitry that controls epigenetic and transcription patterns in esophageal squamous cell Carcinoma Cell lines. Gastroenterology.

[209] Anzalone AV, Randolph PB, Davis JR, Sousa AA, Koblan LW, Levy JM, Chen PJ, Wilson C, Newby GA, Raguram A, Liu DR (2019). Search-and-replace genome editing without double-strand breaks or donor DNA. Nature.

[210] Ye B, Fan D, Xiong W, Li M, Yuan J, Jiang Q, Zhao Y, Lin J, Liu J, Lv Y (2021). Oncogenic enhancers drive esophageal squamous cell carcinogenesis and metastasis. Nat Commun.

[211] Huang G, Zhang X, Xu Y, Chen S, Cao Q, Liu W, Fu Y, Jia Q, Shen J, Yin J, Zhang J (2024). Prognostic and predictive value of super-enhancer-derived signatures for survival and lung metastasis in osteosarcoma. J Transl Med.

[212] Huang P, Zhang B, Zhao J, Li MD (2022). Integrating the Epigenome and Transcriptome of Hepatocellular Carcinoma to identify systematic enhancer aberrations and establish an aberrant enhancer-related prognostic signature. Front Cell Dev Biol.

[213] Gao Y, Zhang T, Terai H, Ficarro SB, Kwiatkowski N, Hao MF, Sharma B, Christensen CL, Chipumuro E, Wong KK (2018). Overcoming resistance to the THZ Series of Covalent Transcriptional CDK inhibitors. Cell Chem Biol.

